# EIF4A3-induced circTOLLIP promotes the progression of hepatocellular carcinoma via the miR-516a-5p/PBX3/EMT pathway

**DOI:** 10.1186/s13046-022-02378-2

**Published:** 2022-05-05

**Authors:** Yachong Liu, Jia Song, Hongwei Zhang, Zhibin Liao, Furong Liu, Chen Su, Weijian Wang, Mengzhen Han, Lu Zhang, He Zhu, Zhanguo Zhang, Huifang Liang, Lei Zhang, Bixiang Zhang, Xiaoping Chen

**Affiliations:** 1 Hepatic Surgery Center, Tongji Hospital, Tongji Medical College, Huazhong University of Science and Technology, 1095 Jiefang Avenue, 430030 Wuhan, Hubei People’s Republic of China; 2 Hubei Key Laboratory of Hepato-Pancreato-Biliary Diseases, Wuhan, Hubei People’s Republic of China; 3 Department of Hepatobiliary Surgery, Shanxi Bethune Hospital, Shanxi Academy of Medical Sciences, Shanxi Medical University; Shanxi Tongji Hospital, Tongji Medical College, Huazhong University of Science and Technology, Taiyuan, 030032 China; 4 Key Laboratory of Organ Transplantation, Ministry of Education, Wuhan, Hubei People’s Republic of China; 5 Key Laboratory of Organ Transplantation, National Health Commission, Wuhan, Hubei People’s Republic of China; 6 Key Laboratory of Organ Transplantation, Chinese Academy of Medical Sciences, Wuhan, Hubei People’s Republic of China

**Keywords:** circTOLLIP, HCC, PBX3, EMT

## Abstract

**Background:**

Circular RNAs (circRNAs) function as crucial regulators in multiple cancers, including hepatocellular carcinoma (HCC). However, the roles of circRNAs in HCC remains largely unknown.

**Methods:**

circTOLLIP was identified in HCC by screening of two public circRNA microarray datasets and detected in HCC cells and tissues through quantitative real-time PCR (qRT–PCR) and in situ hybridization (ISH). Gain- and loss-of-function assays were performed to confirm the biological effects of circTOLLIP on HCC in vitro and in vivo. Mechanistically, bioinformatics analysis of online databases, MS2-RNA pulldown, biotin-labeled circTOLLIP/miR-516a-5p RNA pulldown, RNA immunoprecipitation (RIP), luciferase reporter assay, fluorescence in situ hybridization assay (FISH) and RNA sequencing were used to confirm the regulation of Eukaryotic initiation factor 4A3 (EIF4A3) on circTOLLIP and the interaction among circTOLLIP, miR-516a-5p and PBX homeobox 3 (PBX3).

**Results:**

circTOLLIP was significantly upregulated in HCC cells and tissues. High circTOLLIP expression was correlated with poor overall survival (OS) and disease-free survival (DFS) in patients. circTOLLIP promoted the proliferation and metastasis of HCC cells in vitro and in vivo. Mechanistically, EIF4A3 promoted the biogenesis of circTOLLIP without affecting its stability. Moreover, circTOLLIP sponged miR-516a-5p to elevate the expression of PBX3, thereby activating the epithelial-to-mesenchymal transition (EMT) pathway and facilitating tumor progression in HCC.

**Conclusions:**

Our findings indicate that EIF4A3-induced circTOLLIP promotes the progression of HCC through the circTOLLIP/miR-516a-5p/PBX3/EMT axis.

**Supplementary Information:**

The online version contains supplementary material available at 10.1186/s13046-022-02378-2.

## Background

Primary liver cancer is currently the sixth most commonly diagnosed cancer and the third leading cause of cancer mortality worldwide [1]. As the major type of primary liver cancer, hepatocellular carcinoma accounts for 75-85% of all cases [1]. HCC is characterized by a dismal prognosis and high recurrence rate, which are related to multiple etiological factors, late diagnosis and the high rates of metastasis [2]. The diagnosis of HCC is generally dependent on noninvasive means, such as serum alpha-fetoprotein and diagnostic imaging [3] and the tumor status in HCC is difficult to determine with a single biomarker [4]. In terms of treatment, hepatic resection and liver transplantation are main curative strategies [2]. For early-stage and intermediate-stage HCC, local ablation and transarterial chemoembolization (TACE) are the main treatments [5]. Notably, molecular therapies have been developed as the main complementary treatment for HCC, particularly advanced HCC; these therapies include tyrosine kinase inhibitors, immune-checkpoint inhibitors and monoclonal antibodies [6]. Although the currently available molecular therapies have been demonstrated to improve clinical outcomes, the improvement in median overall survival is still unsatisfactory in patients with advanced stage HCC. Therefore, the molecular mechanism of HCC pathogenesis still needs to be explored to identify new potential diagnostic and therapeutic targets for HCC.

Circular RNAs, a novel class of non-coding RNAs, are generated through back-splicing of precursor mRNAs [7, 8]. Since the first report of circRNA in 1976, circRNAs have been found to be widely expressed in mammals and generally localized in the cytoplasm [9–11]. CircRNAs are structural covalently closed loop without a 5′ capping or a 3′ polyadenylated tail, and they display an extremely stable state and resist digestion by RNA exonucleases [12–14]. As reported, circRNAs participate in various process of biological and pathological progress, including tumorigenesis and progression [15]. CircRNAs are involved in multiple molecular mechanisms, including microRNA (miRNA) and protein sponges or decoys, enhancer of protein function, protein scaffolding, protein recruitment and templates for translation [14]. Recent evidence shows that circRNAs mainly act as miRNA sponge, which modulates the activity of miRNAs through their binding sites, i.e., miRNA response elements (MREs) [16]. For example, circASAP1 has been reported to act as a competing endogenous RNA (ceRNA) by regulating miR-326/miR-532-5p-MAPK1 signaling and promoting HCC cell proliferation and invasion [17]. Therefore, circRNAs may be crucial for further understanding the molecular pathogenic mechanisms.

In the present study, we investigated the expression profiles of circRNAs in HCC tissues by analysis of microarray datasets from the Gene Expression Omnibus (GEO) database and identified a novel abnormally expressed circRNA termed circTOLLIP (circBase ID: hsa_circ_0008301), which is derived generated from exons of Toll-interacting protein (TOLLIP). CircTOLLIP was demonstrated to be upregulated in HCC tissues compared with adjacent nontumor tissues. Further investigations revealed that the overexpression of circTOLLIP promoted HCC cell proliferation and metastasis via sponging of miR-516a-5p to upregulate PBX3 and finally activate the EMT pathway. Our study indicates that circTOLLIP has promising prognostic potential and may be a therapeutic target in HCC patients.

## Methods

### Bioinformatic analysis

HCC circRNA expression profile data were downloaded from the Gene Expression Omnibus database (https://www.ncbi.nlm.nih.gov/geo/). Two circRNA microarray datasets were analyzed: GSE78520, containing 3 liver tumor tissues and matched adjacent nontumor tissues; and GSE97332, containing 7 HCC and matched non-tumor tissues. miRNAs binding to circTOLLIP were predicted with the CircInteractome tool (https://circinteractome.nia.nih.gov/).

### Patient samples

Human liver tumor tissues and corresponding adjacent normal tissues were obtained from patients who underwent liver resection between 2012 and 2015 at the Hepatic Surgery Center, Tongji Hospital, Tongji Medical School, Huazhong University of Science and Technology (HUST; Wuhan, China). This study was approved by the Medical Ethics Committee of Tongji Hospital, and all procedures met the criteria of the Declaration of Helsinki. Written informed consent for specimens was obtained from all patients.

### Plasmids and cell transfection

The circTOLLIP overexpression plasmid (pLent-EIF1a-circTOLLIP-CMV-RFP-P2A-Puro) and control vector were purchased from Vigene Biosciences (Shandong, China). The PBX3 expression vector was constructed by inserting the PBX3 coding region into *XbaI*–*BamHI* sites in 3 × FLAG-pLenti-CMV-GFP-Puro.

The dual-luciferase reporter vector psiCHECK™-2 Vector was purchased from Promega (C8021, USA) and psiCHECK-2-WT-circTOLLIP, psiCHECK-2-MUT-circTOLLIP, psiCHECK-2-WT-PBX3, and psiCHECK-2-MUT-PBX3 were further constructed by Tsingke Biological Technology (Beijing, China). All sequences were verified by DNA Sanger sequencing.

All small interfering RNAs (siRNAs), miR-516a-5p mimic and inhibitor, and the NC mimic and NC inhibitor were synthesized by RiboBio (Guangzhou, China). The miR-516a-5p overexpressing (GV309-hsa-miR-516a) and control lentivirus were purchased from Vigene Biosciences (Shandong, China). Lipofectamine 3000 (Invitrogen) was used for transfection of siRNAs and plasmids into HCC cell lines according to the manufacturer’s protocol. PEI (BIOHUB, Shanghai, China) was used for transfection of plasmids into HEK293T cells.

The target sequences of the siRNAs are listed as follows:

si-hsa_circ_0008301, 5′-GCCCATCACAGGTGTACAT-3′; si-hsa_circ_0055033, 5′-CATATGTGCAGGAGCTGGC-3′; si-hsa_circ_0072088, 5′-GATTTCCAAGCTGGCCCT-3′; si-hsa_circ_0001955, 5′-TTCGAAATCAGGTGAAGGT-3′; si-PBX3_001, 5′-GGAGGTTCTTCAGATAACT-3′; si-PBX3_002, 5′-GGGTTTCAGGTCCTGAGAA-3′; si-PBX3_003, 5′-GCCAAATTGACCCAGATCA-3′; si-EIF4A3_001, CGAGCAATCAAGCAGATCA; si-EIF4A3_002, GCTGGATTACGGACAGCAT. The sequences of microRNA mimic and inhibitor are listed as follows: miR-516a-5p mimic, 5′-UUCUCGAGGAAAGAAGCACUUUC-3′ and 3′-AAGAGCUCCUUUCUUCGUGAAAG-5′; miR-516a-5p inhibitor, 5′-GAAAGUGCUUCUUUCCUCGAGAA-3′; mimic NC, 5′-UUUGUACUACACAAAAGUACUG-3′ and 3′-AAACAUGAUGUGUUUUCAUGAC-5′; inhibitor NC, 5′-CAGUACUUUUGUGUAGUACAAA-3′.

### Fluorescence in situ hybridization (FISH)

A Cy3-labeled probe specific for circTOLLIP (5′-CY3-CTGCGGGAGCTCACCGATGTACACCTGTGATGGGCACATAGCCAACGC-3′) and a FAM-labeled miR-516a-5p probe (5′-FAM-GAAAGUGCUUCUUUCCUCGAGAA-3′) were used for hybridization in HCC cells. The FISH assay was performed using a Fluorescent In Situ Hybridization Kit (RiboBio, China) following the manufacturer’s protocols. Nuclei were counterstained with DAPI. Images were acquired with a laser scanning confocal microscope (LSM710, Carl Zeiss, Germany).

### RNA immunoprecipitation (RIP)

RIP assay was performed to enrich Argonaute 2 (AGO2)- or EIF4A3-bound RNA using Magna RIP™ RNA-Binding Protein Immunoprecipitation Kit (Millipore, Germany). The antibodies used for RIP assays included antibodies against AGO2 (Abcam, UK), EIF4A3 (Abcam), and rabbit/mouse IgG (Millipore). qRT–PCR was subsequently performed to analyze the enriched RNA.

### MS2-FLAG RNA immunoprecipitation

A 6 × MS2 stem–loop sequence (ACATGAGGATCACCCATGT) was inserted into the pcDNA3.1+ vector to construct the pcDNA3.1-6 × MS2 plasmid. The circTOLLIP sequence and its up- or downstream sequences were inserted into the pcDNA3.1-6 × MS2 plasmid between the *EcoRI* and *XbaI sites*. MS2-FLAG and pcDNA3.1-6 × MS2 plasmids were cotransfected into HEK293 cells. Cells were harvested 48 h later, and the lysate was centrifuged at 12000×g for 15 min at 4 °C. After preprecipitation with 50 μl protein A/G-agarose (Santa Cruz, USA) at 4 °C for 2 h, the supernatant was incubated with an anti-Flag (Sigma, USA) antibody with rotation at 4 °C, overnight. Then, 50 μl of protein A/G-agarose was added and incubated for 2 h. The beads were then washed, and the precipitated proteins were obtained for further Western blot analysis.

### Biotin-labeled RNA pulldown

A biotin-labeled circTOLLIP probe (5′-Biotin- CTGCGGGAGCTCACCGATGTACACCTGTGATGGGCACATAGCCAACGC-3′) was synthesized by Sangon Biotech (Shanghai, China). A biotin-labeled-miR-516a-5p probe (5′-FAM-GAAAGTGCTTCTTTCCTCGAGAA-3′) was synthesized by RiboBio. Biotin-labeled RNA pulldown was performed using an RNA Antisense Purification (RAP) Kit (BersinBio, Guangzhou, China) according to the manufacturer’s instructions. The enriched RNA was detected by qRT–PCR.

### Statistical analysis

All statistical analyses were performed using SPSS 21.0 or GraphPad Prism 8.0 software. Data are presented as the mean ± standard deviation (SD) values. Continuous variables were compared with the two-tailed Student’s t test. Categorical data were analyzed by Pearson’s χ2 test or Fisher’s exact test. Correlations were analyzed by calculating the Pearson correlation coefficient. Survival analysis was performed according to the Kaplan–Meier method with the log-rank test. A *P* value<0.05 was considered significant (*P < 0.05; **P < 0.01; ***P < 0.001; NS, nonsignificant).

The other detailed methods can be found in the Supplementary Methods.

## Results

### Identification and characterization of circTOLLIP in HCC

**Fig. 1:**
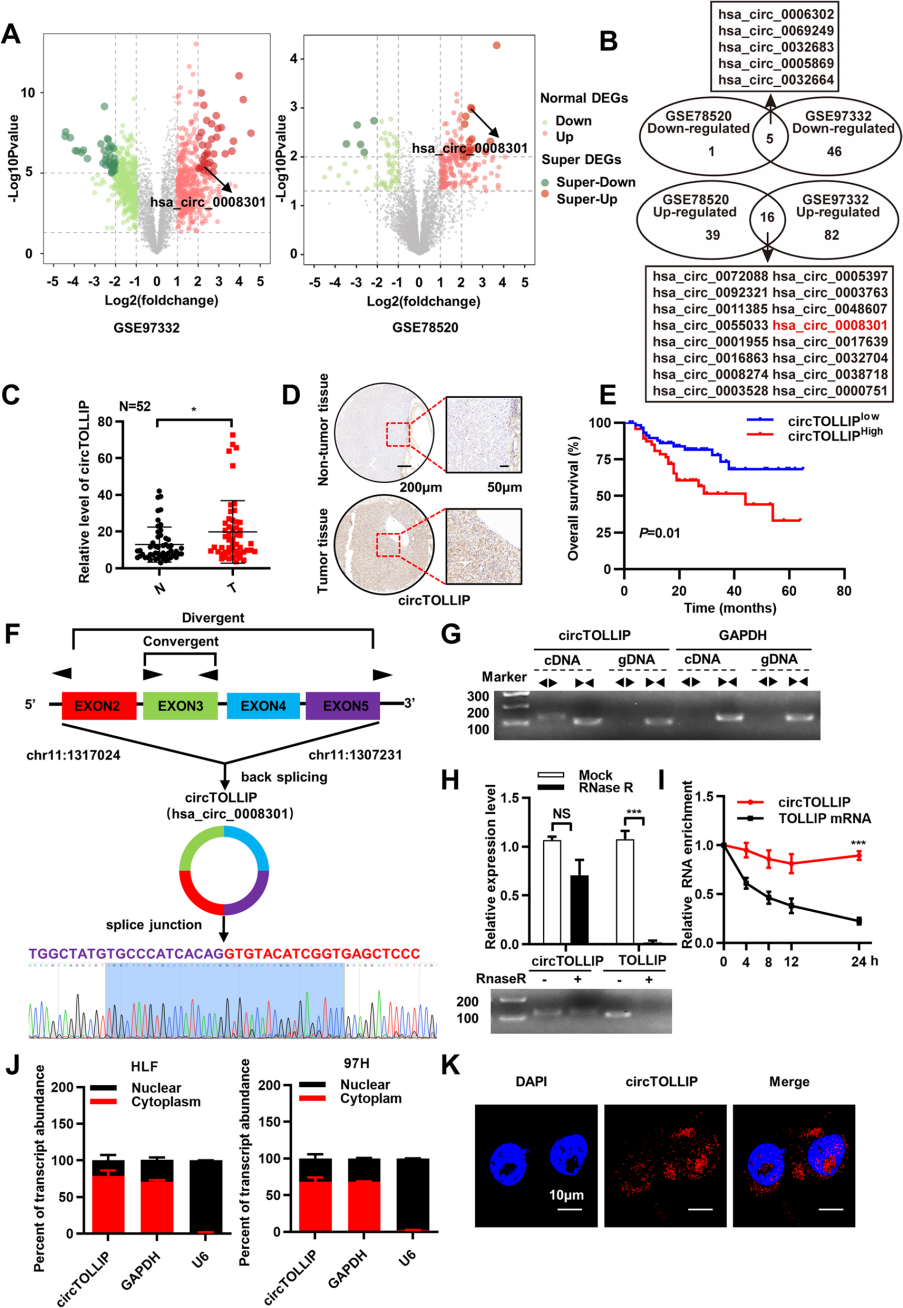
Identification and characterization of circTOLLIP in HCC. **a** Volcano plot of circRNAs from GEO datasets of GSE97332 and GSE78520. Significantly upregulated and downregulated circRNAs are separately denoted in red and green. **b** Venn diagram showing the overlap between the two datasets. CircRNAs with *P* < 0.01 and |log2(foldchange)| ≥ 2 were chosen. **c** qRT–PCR analysis of the circTOLLIP expression in tissues. T, tumor tissues; N, adjacent nontumor tissues. **d** Representative ISH images of circTOLLIP in microarrays containing 138 paired HCC specimens. **e** Kaplan–Meier curve of the correlation between circTOLLIP expression and overall survival (OS). low circTOLLIP group: *n* = 63, high circTOLLIP group: *n* = 49. **f** Schematic display of circTOLLIP formation and the principle of divergent and convergent primer design. The back-splicing junction sites were confirmed by Sanger sequencing. **g** PCR amplification of circTOLLIP and its linear isoform using divergent and convergent primers from cDNA and genomic DNA (gDNA). GAPDH was used as control. **h** qRT–PCR analysis of circTOLLIP and TOLLIP mRNA levels with or without RNase R treatment. **i** Stability of circTOLLIP and TOLLIP RNA with or without actinomycin D treatment at the specific time point measured by qRT–PCR. **j** The RNA level of circTOLLIP in the nucleus and cytoplasm of HLF and 97H cells. **k** The subcellular location of circTOLLIP was validated mainly in the cytoplasm by using a FISH assay. Nuclei, blue; circTOLLIP, red. Scale bars = 10 μm

To identify differentially expressed circRNAs in HCC, we analyzed two GEO datasets (GSE97332 and GSE78520). The GSE97332 dataset was generated from seven human HCC tumor and matched adjacent nontumor tissues [18]. GSE78520 contains data from non-coding RNA profiling of three pair human liver cancer and adjacent normal liver tissues [19]. All differentially expressed circRNAs that met the criteria of |log2(foldchange)| ≥ 2 and *P*<0.01 were included in the analysis and shown in the volcano plot (Fig. 1a). Among these circRNAs, the 16 most upregulated circRNAs overlapped in the two datasets (Fig. 1b). qRT–PCR was performed to investigate the relative abundances of these circRNAs in four HCC cell lines (Hep3B, Huh7, HLF, and 97H), and we considered highly expressed circRNAs (hsa_circ_0008301, hsa_circ_0055033, hsa_circ_0072088, and hsa_circ_0001955) as more likely candidates (Additional file 1: Fig. S1a). Then, a Cell Counting Kit-8 (CCK-8) assay was conducted to assess the proliferation-related biological effects in HLF and 97H cells transfected with the corresponding siRNAs. The results showed that downregulation of hsa_circ_0008301 markedly inhibited the growth of HLF and 97H cells, while knockdown of hsa_circ_0055033, hsa_circ_0072088 and hsa_circ_0001955 showed no significant difference in their proliferative effects on HLF and 97H cells (Additional file 1: Fig. S1b-c). And we found that the expression of circTOLLIP (circBase ID: hsa_circ_0008301) was significantly higher in 75% (39 of 52) of the HCC tissues than in the corresponding adjacent nontumor tissues (Fig. 1c; Additional file 1: Fig. S1d). Higher level of circTOLLIP in HCC specimens was further found by ISH staining of 138 paired HCC samples compared to nontumor tissues (Fig. 1d; Additional file 1: Fig. S1f). Moreover, Kaplan–Meier survival analysis showed that a high level of circTOLLIP was associated with the poor overall survival and disease-free survival in HCC patients (Fig. 1e; Additional file 1: Fig. S1g). In addition, the relative expression level of circTOLLIP was higher in highly malignant HCC cells, such as in 97H and LM3 cells (Additional file 1: Fig. S1e).

CircTOLLIP is derived from exons 2-5 of TOLLIP pre-mRNA and has a length of 577 nt. The back-spliced junction was detected using divergent primers and was then validated by Sanger sequencing, which is consistent with the annotation in circBase (http://www.circbase.org/) (Fig. 1f). PCR analysis demonstrated that circTOLLIP could be amplified by divergent primers only from cDNA (reverse-transcribed RNA) and not from gDNA (genomic DNA), while linear TOLLIP products could be amplified by convergent primers from both cDNA and gDNA (Fig. 1g). Notably, circTOLLIP was more resistant to RNase R digestion and had a longer half-life than linear TOLLIP mRNA treated with Actinomycin D, revealing that circTOLLIP is more stable than linear TOLLIP (Fig. 1h-i). qRT–PCR analysis of RNA in the nuclear and cytoplasmic fractions was performed to confirm the cellular localization of circTOLLIP, and results showed that circTOLLIP was predominantly localized in the cytoplasm (Fig. 1j). A FISH assay was conducted with a CY3-labeled circTOLLIP probe, and this assay also revealed the predominant cytoplasmic location of circTOLLIP in HCC cells (Fig. 1k).

Collectively, the above results confirm the characteristics of circTOLLIP as a novel circRNA. These findings also suggest that circTOLLIP may play an important role in the pathogenesis of HCC and thereby function as a potential therapeutic target in HCC.

### CircTOLLIP promotes the proliferation and metastasis of HCC cells in vitro and in vivo

To further investigate the biological roles of circTOLLIP in HCC progression, we constructed siRNA targeting the back-splicing junction sites (Additional file 2: Fig. S2a) and a vector for ectopic overexpression of circTOLLIP. As expected, circTOLLIP was successfully downregulated and overexpressed by the siRNA and overexpression vector, respectively, in HLF and 97H cells (Additional file 2: Fig. S2b-c); however, the TOLLIP mRNA level showed no significant changes (Additional file 2: Fig. S2d-e).

**Fig. 2:**
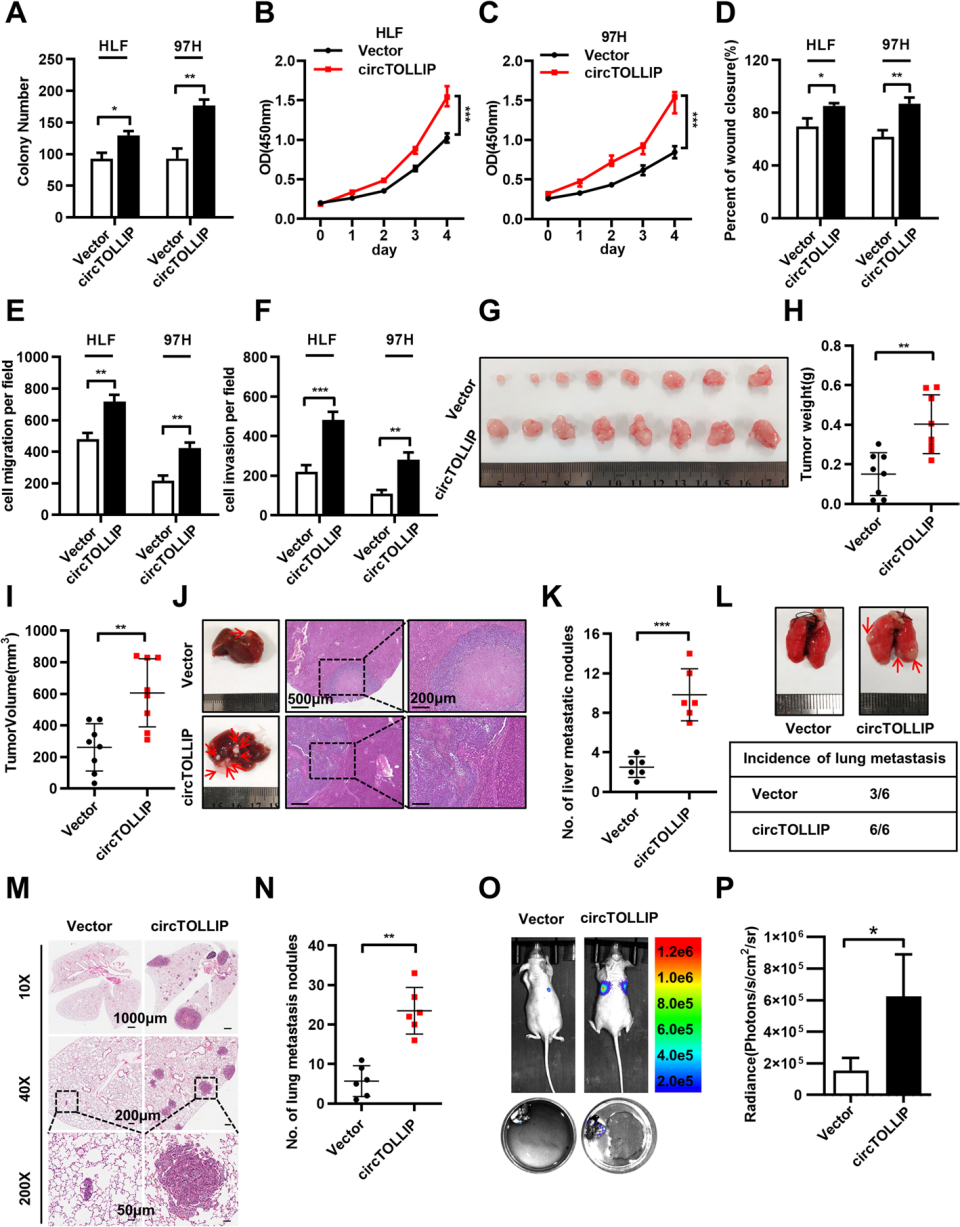
circTOLLIP promotes the proliferation and migration of HCC cells in vitro and in vivo. **a-c** The colony formation assay and CCK-8 assay were performed in HLF and 97H cells. **d-f** The statistic graphs of scratch wound healing assay and cell migration and invasion assays. **g** The subcutaneous xenograft tumors from vector or overexpressed circTOLLIP 97H cells. **h-i** Tumor weight and tumor volume of subcutaneous xenografts. **j-k** Liver orthotopic transplantation and metastasis models in nude mice with circTOLLIP-overexpressing and vector HLF cells. Representative pictures are presented that show HE staining of isolated liver tissue and a general image of the whole liver. The number of visible tumor nodules was counted and pointed by red arrows. **l-p** Lung metastasis assay was performed with 97H-circTOLLIP cells in nude mice by tail vein injection. Representative pictures showing lung metastatic nodules, the incidence of lung metastasis (**l**), HE staining of lung tissue (**m**) and lung fluorescence imaging (**o, p**) were presented. The number of lung metastatic nodules was counted in lung tissues after HE staining(**n**)

The cell colony formation assay and CCK-8 assay revealed that the overexpression of circTOLLIP promoted the growth of HLF and 97H cells and that downregulation of circTOLLIP reduced cell viability (Fig. 2a-c; Additional file 2: Fig. S2f-i). The scratch wound healing assay and Transwell migration and invasion assays displayed that overexpression of circTOLLIP promoted the migration and invasion ability of HLF and 97H cells, while downregulation of circTOLLIP produced the opposite effects (Fig. 2d-f; Additional file 2: Fig. S2j-l; Additional file 3: Fig. S3a-b).

To further explore the effects of circTOLLIP in vivo, we established subcutaneous xenograft model, orthotopic xenograft tumor metastasis model and pulmonary metastasis model. To establish the subcutaneous xenograft model, stably transfected 97H-vector or 97H-circTOLLIP cells were injected into the axillae of nude mice (eight mice in each group). The results revealed that circTOLLIP was stably overexpressed in 97H-circTOLLIP xenograft tumors (Additional file 4: Fig. S4a). Moreover, the tumor size was larger in the 97H-circTOLLIP group than in the vector group, and the average tumor weight and tumor volume were significantly higher in the 97H-circTOLLIP group than in the vector group (Fig. 2g-i). Consistent with these results, in the orthotopic xenograft tumor metastasis model, we found that the mice in the circTOLLIP overexpression group emerged more and larger intrahepatic tumor metastatic nodules than the mice in the vector group (Fig. 2j-k; Additional file 4: Fig. S4b-c). To establish the pulmonary metastasis model, we injected 97H-luciferase-vector cells or 97H-luciferase-circTOLLIP cells into nude mice via the tail vein (six mice per group). Results showed that 100% (6/6) of the mice in the 97H-luciferase-circTOLLIP group exhibited metastatic lung tumor nodules, while metastatic lung tumor nodules were ultimately found in only 50% (3/6) of mice in the 97H-luciferase-vector group; in addition, the number of tumor nodules was greater in the 97H-luciferase-circTOLLIP group. The tumor size was larger in mice injected with circTOLLIP-overexpressing cells than in mice injected with 97H-luciferase-vector cells (Fig. 2l-n). Moreover, in vivo luciferase activity was higher in mice injected with circTOLLIP-overexpressing cells than in mice injected with 97H-luciferase-vector cells (Fig. 2o-p; Additional file 4: Fig. S4d-e).

According to the above results, circTOLLIP could promote the proliferation and metastasis of HCC cells in vitro and in vivo.

### EIF4A3 promotes the biogenesis of circTOLLIP

EIF4A3 is a member of the EIF4A DEAD-box helicase family of translation initiation factors. Many RNA-binding proteins have been reported to regulate the biogenesis of circRNAs. QKI [20] and FUS [21] were reported to regulate circRNA biogenesis by binding the up- or downstream introns flanking the back-splicing junctions. EIF4A3 has been reported [22] to be a novel component of the exon junction complex and could also promote the formation of circMMP9 [23]. We therefore explored whether EIF4A3 is involved in the biogenesis of circTOLLIP.

**Fig. 3:**
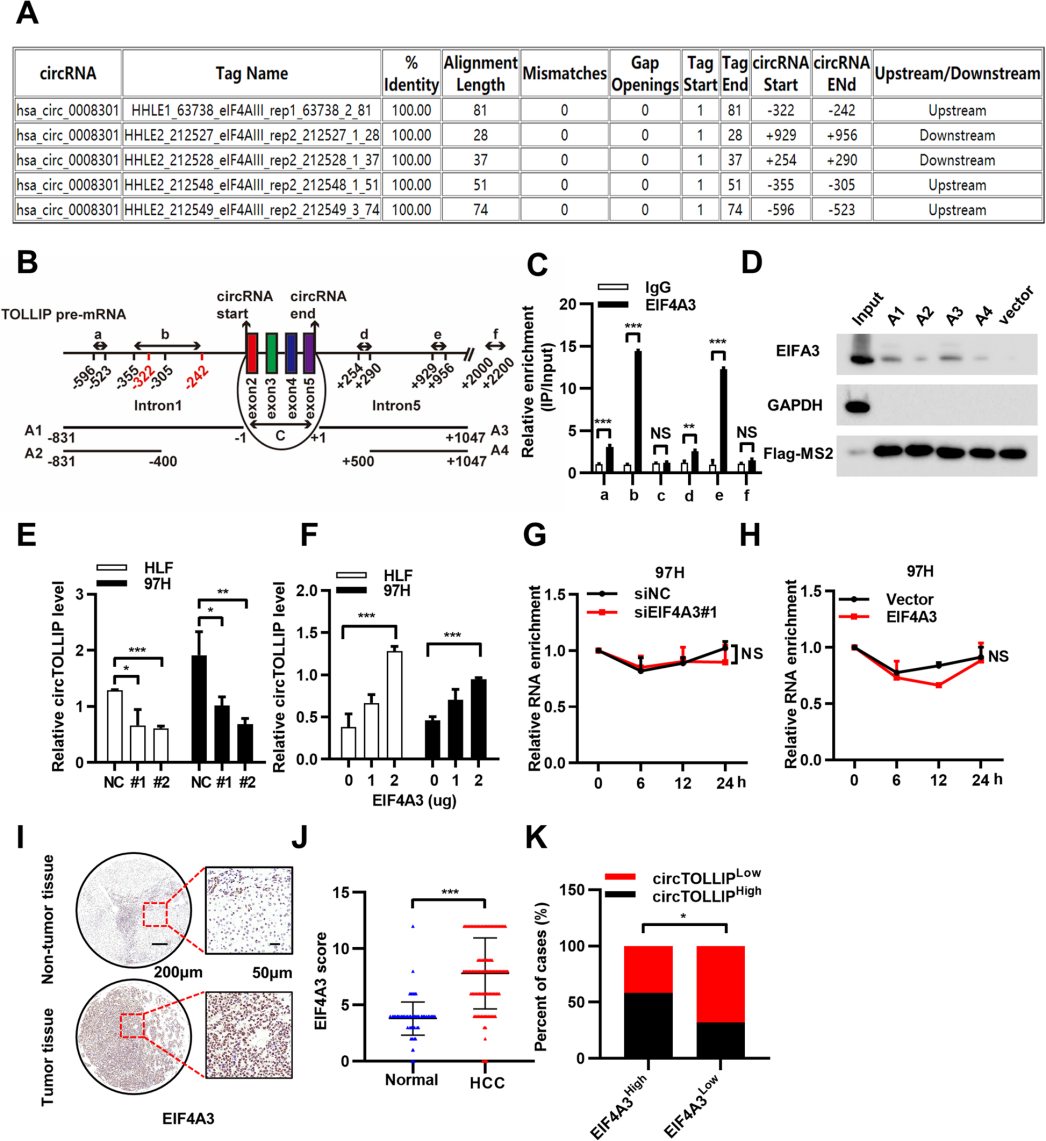
EIF4A3 promotes the biogenesis of circTOLLIP. **a** Prediction of the putative EIF4A3 binding sites in TOLLIP pre-mRNA by CircInteractome database. **b** Six positions (a-f) in TOLLIP pre-mRNA were selected to design qPCR primers and 4 plasmids (A1-A4) containing EIF4A3 binding sites were constructed to pull down EIF4A3 protein. **c** RIP assay using EIF4A3 antibody verified the direct binding of EIF4A3 and TOLLIP pre-mRNA. **d** Western blot analysis of EIF4A3 protein with MS2-RNA pulldown assay. Vector and GAPDH were used as negative controls. **e-f** qRT–PCR analysis of circTOLLIP level in HLF and 97H cells with transfection of EIF4A3 siRNA or EIF4A3 overexpression plasmid. **g-h** Stability of circTOLLIP in EIF4A3-overexpressing or EIF4A3 knockdown 97H cells with actinomycin D treatment. **i** Representative images of IHC staining in 138 pairs of HCC tissues. **j** Statistical analysis of the IHC results. **k** Correlation analysis between circTOLLIP and EIF4A3 expression in HCC tumors

We conducted bioinformatics analysis with the CircInteractome tool (https://circinteractome.nia.nih.gov/rna_binding_protein.html) and found three putative EIF4A3 binding sites in the upstream and two putative binding sites in the downstream of circTOLLIP pre-mRNA (Fig. 3a). Due to the overlap of the two binding sites in intron 1, we named the putative binding sequences separately as *a* and *b*. And we named the sequence on circTOLLIP as *c*, the sequences on intron 5 as *d*, *e* and a random sequence on intron 5 as *f* (Fig. 3b). A RIP assay was performed with an anti-EIF4A3 antibody. Results showed that EIF4A3 could bind to *a*, *b*, *d*, *e* but not c and *f* (Fig. 3c), which indicated that EIF4A3 formed a protein-RNA complex via the putative binding sites in TOLLIP pre-mRNA. Four plasmids were constructed containing MS2 binding sites and the up- or downstream EIF4A3 binding sites of circTOLLIP, which named respectively as A1, A2, A3 and A4 (Fig. 3b). An MS2 RNA pulldown assay was subsequently performed and confirmed that EIF4A3 could bind to both the upstream and downstream sequences of circTOLLIP (Fig. 3d). Knockdown of EIF4A3 protein reduced the circTOLLIP RNA level in HLF and 97H cells (Fig. 3e; Additional file 5: Fig. S5a), while the mRNA level of TOLLIP showed no significant change (Additional file 5: Fig. S5b). The actinomycin D assay revealed that the stability of circRNA could not be regulated by EIF4A3 (Fig. 3g). At the same time, overexpression of EIF4A3 facilitated the expression of circTOLLIP without changing TOLLIP mRNA level or the stability of circTOLLIP (Fig. 3f and h; Additional file 5: Fig. S5c-d). To further explore the expression of EIF4A3 in HCC patients, an immunohistochemistry assay was conducted in 138 paired HCC tissues and revealed that the expression of EIF4A3 was higher in HCC tissues than in nontumor tissues (Fig. 3i-j) and that the expression of EIF4A3 and circTOLLIP was positively correlated in HCC patients (Fig. 3k). Taken together, these results indicate that EIF4A3 promotes the biogenesis of circTOLLIP.

### circTOLLIP serves as a sponge for miR-516a-5p

**Fig. 4:**
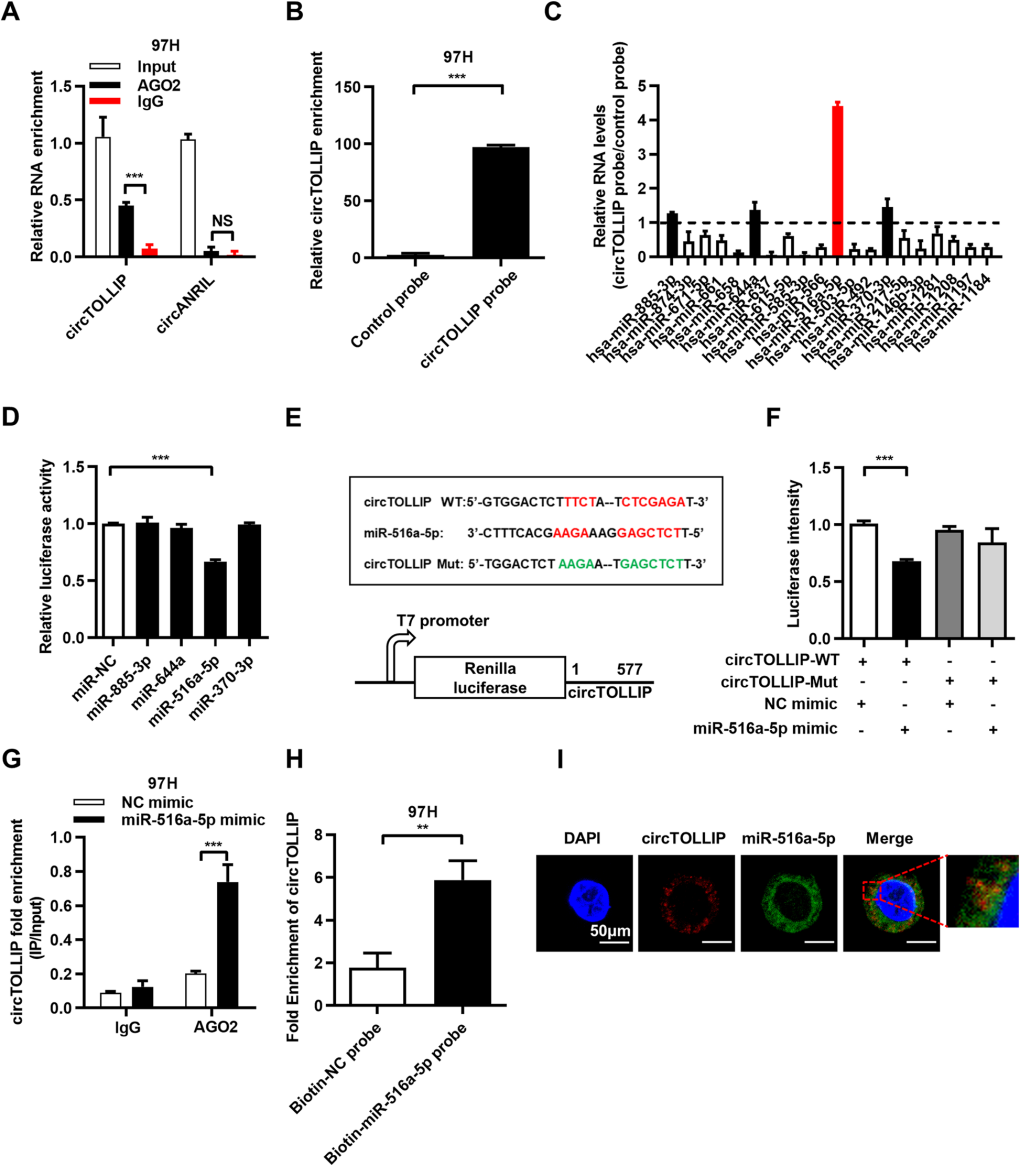
circTOLLIP serves as a sponge for miR-516a-5p. **a** RIP was performed with an anti-AGO2 antibody in 97H cells. **b-c** Relative circTOLLIP and microRNA enrichment using biotinylated control probe and specific circTOLLIP probes in 97H cells. **d** Relative luciferase reporter activity of WT-circTOLLIP after transfection with mimics of the most 4 most enriched microRNAs in HEK-293 T cells. **e** Schematic view of miR-516a-5p putative binding sites with circTOLLIP and construction of correspondent mutant circTOLLIP reporter plasmid. **f** Luciferase reporter activity of circTOLLIP in 97H cells co-transfected with miR-516a-5p mimic or NC mimic. **g** RIP was performed using an anti-AGO2 antibody in 97H cells transfected with miR-516a-5p mimic or NC mimic. **h** circTOLLIP enrichment using biotinylated NC probe and miR-516a-5p probes in 97H cells. **i** Colocalization between cicTOLLIP and miR-516a-5p was observed by FISH assay. Scale bars = 50 μm

Given that circRNAs in the cytoplasm have been shown to act as miRNA sponges [16] and that circTOLLIP is predominantly localized in the cytoplasm, we explored whether circTOLLIP is bonded to microRNAs. We first conducted bioinformatic analysis (https://circinteractome.nia.nih.gov/rna_binding_protein.html) and identified two putative AGO2 binding sites on circTOLLIP (Additional file 6: Fig. S6a). To further verify their binding, we performed an RNA immunoprecipitation assay with an anti-AGO2 antibody and found that endogenous circTOLLIP was specifically enriched by AGO2, while circANRIL, a circRNA reported to not bind to AGO2 [24], was not significantly enriched (Fig. 4a; Additional file 6: Fig. S6b).

We next predicted the potential binding microRNAs by with the CircInteractome tool and identified 20 microRNAs that could directly bind to circTOLLIP (Additional file 6: Fig. S6c). An RNA pulldown assay was subsequently performed with a biotin-labeled circTOLLIP probe, and circTOLLIP was successfully enriched in HLF and 97H cells (Fig. 4b; Additional file 6: Fig. S6d). After purifying the enriched RNA, we analyzed the abundances of the 20 microRNAs by qRT–PCR analysis and observed relative enrichment of miR-885-3p, miR-644a, miR-516a-5p, and miR-370-3p (Fig. 4c). To validate the binding capabilities of these miRNAs to circTOLLIP, we constructed a WT-circTOLLIP luciferase reporter plasmid and co-transfected with mimic of the above 4 candidate microRNAs into HEK-293 T cells (Fig. 4e). The luciferase signals in miR-516a-5p group decreased, while transfection of the miR-885-3p, miR-644a and miR-370-3p mimics did not change the luciferase reporter activity (Fig. 4d). In line with the circTOLLIP pulldown results, miR-516a-5p was also the most enriched microRNA, which indicated that among the 20 microRNAs, only miR-516a-5p can directly bind circTOLLIP.

We then constructed a circTOLLIP mutant luciferase reporter plasmid, and found that the luciferase activity of the WT-circTOLLIP reporter was significantly reduced compared with that of mutated circTOLLIP luciferase reporter when co-transfected with miR-516a-5p mimic (Fig. 4e-f). Additionally, by a RIP assay using an anti-AGO2 antibody, we found obvious enrichment of circTOLLIP after transfecting the miR-516a-5p mimic into HLF and 97H cells (Fig. 4g; Additional file 6: Fig. S6e). Furthermore, an RNA pulldown assay with a biotin-labeled miR-516a-5p probe revealed that circTOLLIP was significantly enriched in HLF and 97H cells (Fig. 4h; Additional file 6: Fig. S6f-h). FISH also demonstrated the colocalization of circTOLLIP and miR-516a-5p in the cytoplasm (Fig. 4i).

Furthermore, neither upregulation nor downregulation of circTOLLIP regulated the expression of miR-516a-5p in HLF and 97H cells (Additional file 6: Fig. S6i-j). Consistent with this finding, circTOLLIP did not show significant changes after overexpression or inhibition of miR-516a-5p (Additional file 6: Fig. S6k-l). Collectively, all these results prove that circTOLLIP functions as a sponge of miR-516a-5p.

### miR-516a-5p inhibits the growth of HCC cells in vitro and in vivo

To further clarify whether the biological function of circTOLLIP is connected with miR-516a-5p, we thereby investigated the role of miR-516a-5p in HCC.

**Fig. 5:**
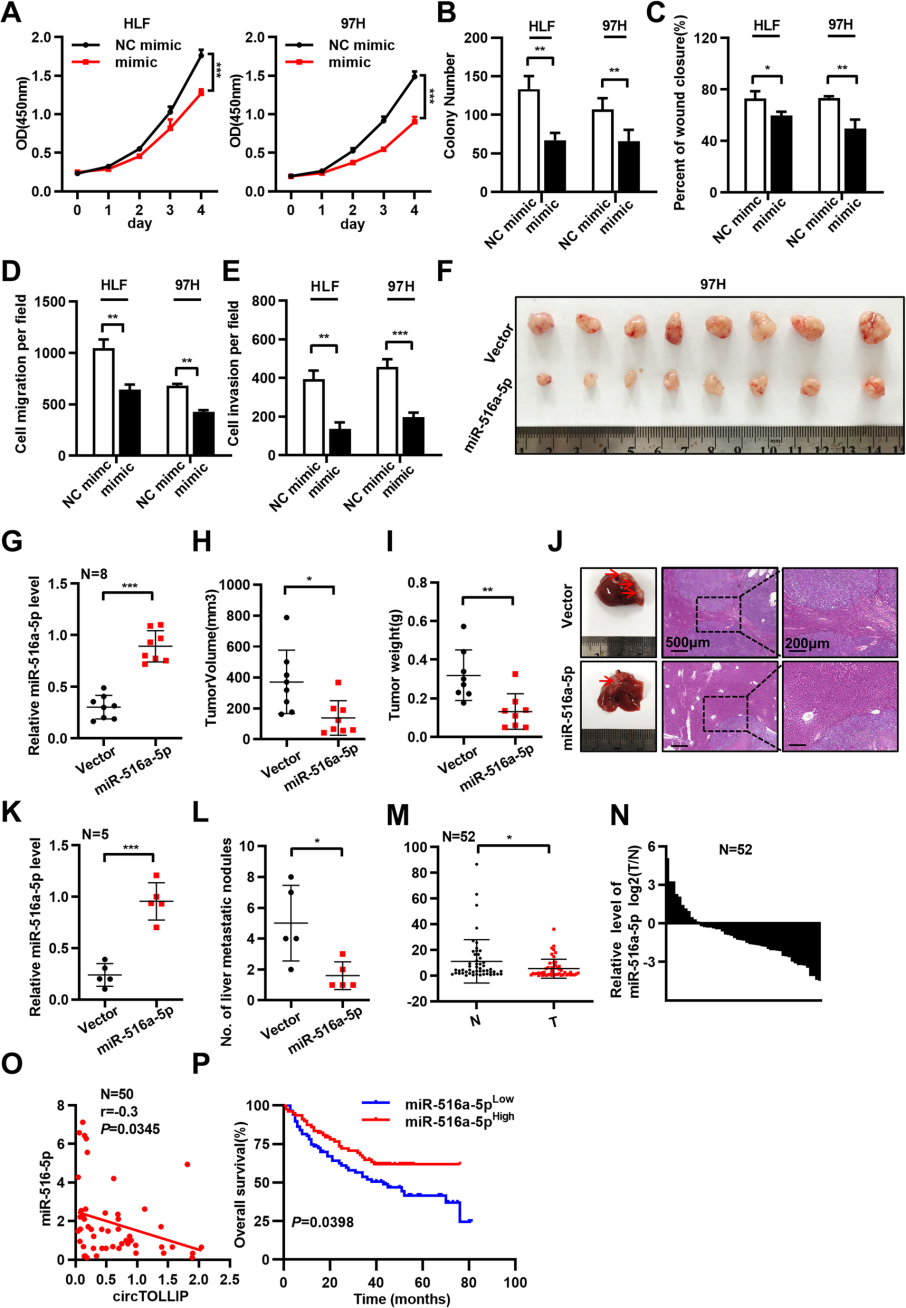
miR-516a-5p inhibits the proliferation and metastasis of HCC cells in vitro and in vivo*.* **a** CCK-8, cell colony formation assay (**b**), scratch wound healing assay (**c**), and cell migration and invasion assays (**d, e**) were performed in HLF and 97H cells transfected with miR-516a-5p mimic or NC mimic. **f** The subcutaneous xenograft tumors from nude mice that transplanted with stablely overexpressing miR-516a-5p and vector 97H cells. **g** qRT–PCR analysis of miR-516a-5p level in xenograft tumors and quantification of tumor volume (**h**) and weight (**i**). **j** Livers from nude mice orthotopically transplanted with miR-516a-5p-overexpressing and vector 97H cells. Representative images showing the livers from the nude mice and HE staining of isolated liver tissues. The miR-516a-5p expression was analyzed in two groups (**k**) and the number of visible tumor nodules was counted (**l**). **m-n** The relative miR-516a-5p levels in 52 paired HCC and adjacent nontumor tissues. **o** The correlation between circTOLLIP and miR-516a-5p in HCC tissues. **p** Kaplan–Meier analysis of the correlation between miR-516a-5p expression and OS in HCC according to data from Kaplan–Meier Plotter database. Low miR-516a-5p group: *n* = 87, high miR-516a-5p group: *n* = 79

We measured the expression level of miR-516a-5p in HCC cells and successfully overexpressed or downregulated it in HLF and 97H cells (Additional file 7: Fig. S7a-c). The CCK-8 assay, colony formation assay, scratch wound healing assay and Transwell migration and invasion assays showed that overexpression of miR-516a-5p attenuated the growth and metastasis capacity of HCC cells, while the inhibition of miR-516a-5p promoted the growth and metastasis of HCC cells (Fig. 5a-e; Additional file 7: Fig. S7d-i; Additional file 8: Fig. S8a-b). To investigate the function of miR-516a-5p in vivo, we established a subcutaneous xenograft model and orthotopic xenograft tumor metastasis model in nude mice. The results showed showed that the growth of subcutaneous tumors was significantly inhibited in mice injected with miR-516a-5p overexpressing cells (Fig. 5f-i). Moreover, there were fewer intrahepatic metastases and smaller tumors in situ in this group than in the control group (Fig. 5j-l). qRT–PCR analysis of 52 paired tumor and adjacent nontumor tissues revealed that miR-516a-5p RNA level was lower in tumor tissues (Fig. 5m-n) and the RNA levels of miR-516a-5p and circTOLLIP were negatively correlated in HCC patients (Fig. 5o). According to the prognosis analysis of Kaplan-Meier Plotter database (http://www.kmplot.com/analysis/index.php?p=service&cancer=liver_rnaseq), the low expression of miR-516a-5p is associated with poor overall survival in HCC patients (Fig. 5p). These results prove that miR-516a-5p inhibits the growth of HCC cells in vitro and in vivo.

### circTOLLIP relieves the repression of miR-516a-5p on PBX3

**Fig. 6:**
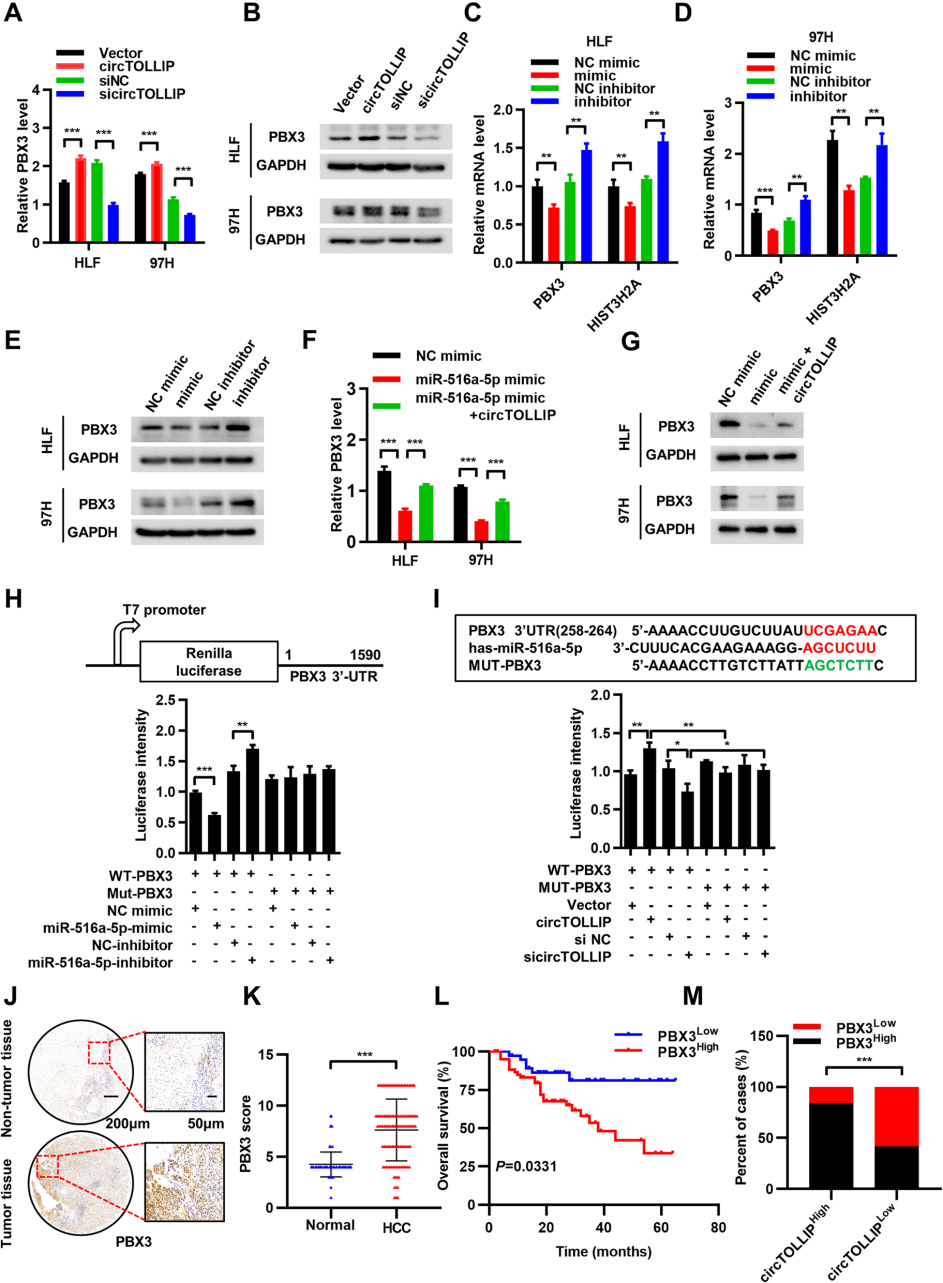
circTOLLIP relieves repression of miR-516a-5p on PBX3. **a-b** PBX3 expression in HLF and 97H cells with circTOLLIP overexpression or knockdown. **c-d** qRT–PCR analysis of PBX3 and HIST3H2A in HLF and 97H cells transfected with miR-516a-5p mimic or inhibitor. **e** PBX3 protein levels in HLF and 97H cells transfected miR-516a-5p mimic or inhibitor. **f-g** PBX3 expression in HLF and 97H cells transfected with miR-516a-5p mimic alone or in combination with circTOLLIP. **h** Luciferase reporter activity of PBX3-3’UTR in 97H cells cotransfected with miR-516a-5p mimic or inhibitor. **i** Luciferase reporter activity of PBX3-3’UTR in 97H cells with circTOLLIP overexpression or knockdown. **j** Representative images of PBX3 IHC staining in 138 pairs of HCC tissues and **s**tatistical analysis of the IHC scores (**k**). **l** The correlation between PBX3 expression and OS by Kaplan–Meier analysis. Low PBX3 group: *n* = 40, high PBX3 group: *n* = 65. **m** Correlation analysis between circTOLLIP and PBX3 expression in HCC tumors

Next, we explored the targets that could be regulated by the circTOLLIP-miR-516a-5p axis to further elucidate the mechanism of circTOLLIP in HCC. We first analyzed the potential targets of miR-516a-5p in three microRNA databases (TargetScan, DIANA TOOLs and mirDIP). 45 candidate targets were selected, and qRT–PCR was subsequently performed to confirm the specific targets that can be downregulated after miR-516a-5p overexpression in HLF and 97H cells (Additional file 9: Fig. S9a). HIST3H2A was reported to be a direct target of miR-516a-5p in non-small-cell lung cancer (NSCLC) cells [25]. We also verified the downregulation of HIST3H2A by miR-516a-5p in HLF and 97H cells and used HIST3H2A as the positive control to investigate the other targets of miR-516a-5p. Only PBX3 and HIST3H2A were found decreased both in HLF and in 97H cells after transfection with miR-516a-5p mimic (Additional file 9: Fig. S9b-c). Therefore, PBX3 and HIST3H2A were selected as candidate targets of circTOLLIP. We then detected the expression of PBX3 and HIST3H2A after overexpressing or silencing circTOLLIP and found that PBX3, but not HIST3H2A was obviously upregulated after overexpression of circTOLLIP and downregulated after silencing circTOLLIP (Fig. 6a-b; Additional file 9: Fig. S9d). Moreover, inhibition of miR-516a-5p protected PBX3 from downregulation in HLF and 97H cells (Fig. 6c-e). Additionally, circTOLLIP partially rescued PBX3 downregulation in HLF and 97H cells transfected with the miR-516a-5p mimic (Fig. 6f-g).

To further confirm the direct regulation of PBX3 by miR-516a-5p, we constructed a PBX3-3′-UTR luciferase reporter plasmid (WT-PBX3) and a PBX3 mutant luciferase reporter plasmid (Mut-PBX3) in which the predicted miR-516a-5p binding sites in PBX3 were mutated. The luciferase reporter assay revealed that the luciferase activity of only the WT-PBX3 reporter decreased when cotransfected with the miR-516a-5p mimic and increased when cotransfected with the miR-516a-5p inhibitor. However, the Mut-PBX3 reporter had no response to the change in miR-516a-5p expression (Fig. 6h). Moreover, upregulation of circTOLLIP increased the luciferase activity of WT-PBX3 reporter, and transfection of circTOLLIP siRNAs attenuated the luciferase activity of the WT-PBX3 reporter. In addition, the Mut-PBX3 reporter also had no response to the change in circTOLLIP expression (Fig. 6i). PBX3 protein expression was significantly upregulated in HCC tumor tissues (Additional file 9: Fig. S9e), and a high level of PBX3 expression in tumor tissues was also observed via IHC staining in the same microarrays containing 138 paired HCC specimens (Fig. 6j). According to the scores for IHC results, elevated PBX3 expression in HCC tissues was further validated (Fig. 6k). Kaplan–Meier survival analysis showed that HCC patients with higher PBX3 expression had a poorer OS and DFS than those with lower PBX3 expression (Fig. 6l; Additional file 9: Fig. S9f). Moreover, PBX3 expression was positively correlated with circTOLLIP expression (Fig. 6m) in HCC tissues. Taken together, these findings indicate that PBX3 is a direct downstream target of miR-516a-5p and that circTOLLIP relieves the repression of miR-516a-5p on PBX3.

### circTOLLIP stimulates EMT by miR-516a-5p/PBX3 axis

PBX3 is a transcription factor of the pre-B cell leukemia family, which is a group of homeodomain-containing transcription factors and has vital roles in early development [26]. The degradation of PBX3 protein was reported to be independent of the ubiquitin-proteasome system but instead associated with miRNAs in hepatoma cells [27]. Furthermore, PBX3 has been reported to have an oncogenic role in promoting cell proliferation and migration, such as in glioblastoma [28] or colorectal cancer [29]. Notably, PBX3 was reported to be a novel indicator of EMT in colorectal cancer [29], and it was reported to possibly be regulated by multiple miRNAs and to be essential for liver tumor-initiating cells [30].

To explore the role of PBX3 in HCC progression, we constructed HLF and 97H cells stably overexpressing PBX3 and successfully knocked down the PBX3 protein level in HLF and 97H cells transfected with siRNA (Additional file 10: Fig. S10a-b). Upregulation of PBX3 promoted the proliferation and metastasis in HCC cells and knockdown of PBX3 showed opposite biological effects. (Additional file 10: Fig. S10c-i, Additional file 11: Fig. S11a-f, Additional file 12: Fig. S12a-b).

**Fig. 7:**
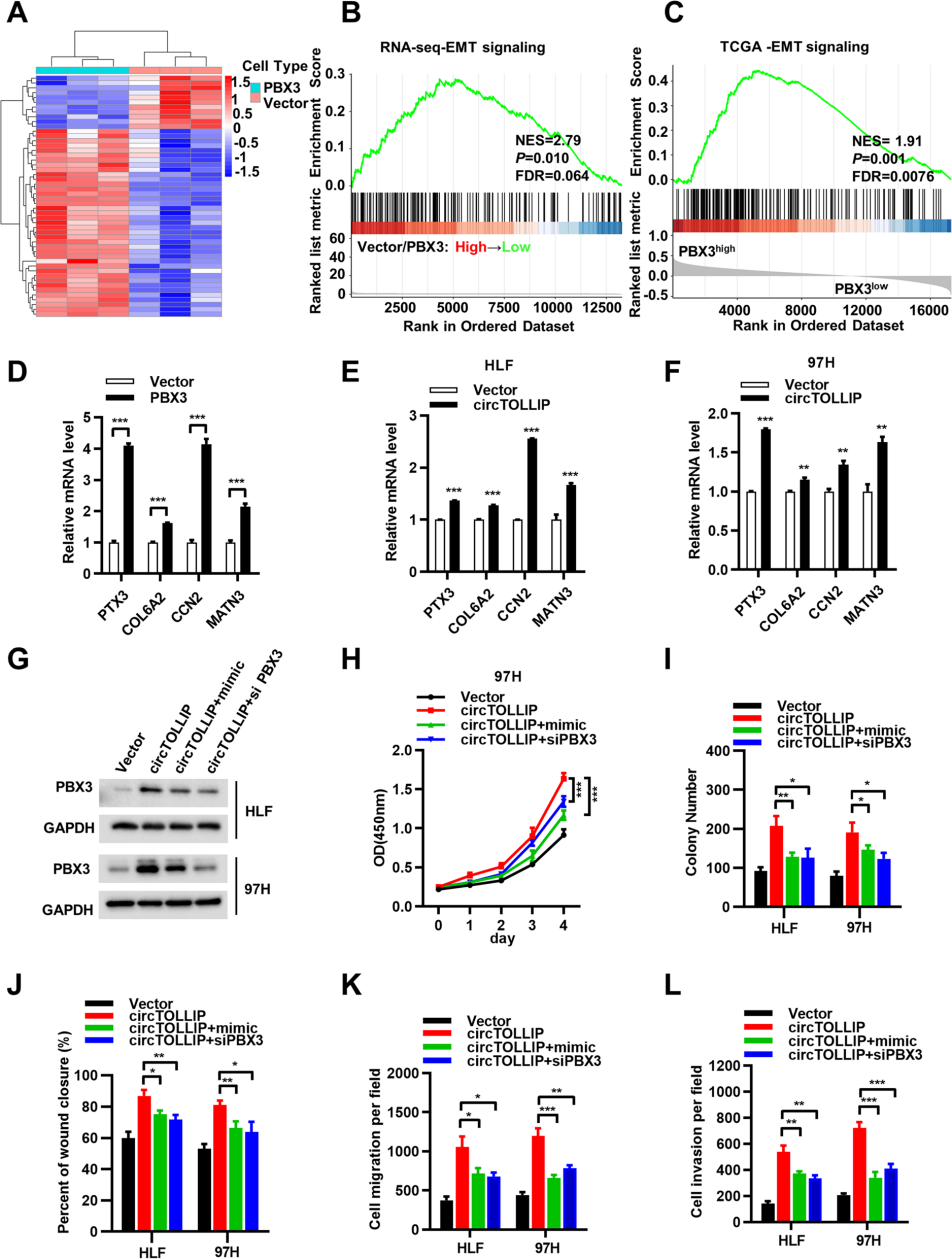
circTOLLIP stimulates EMT by miR-516a-5p/PBX3 axis. **a** Clustering heatmap showing the RNA-seq profiles in vector and PBX3-overexpressing 97H cells. **b-c** Gene set enrichment analysis (GSEA) of genes from RNA-seq profiles and TCGA database comparing with EMT_PATHWAY. **d** Among the EMT-related genes in TCGA, those with the highest positive correlation with PBX3 expression were selected and intersected with the differentially upregulated genes in the RNA-seq profiles. The top four genes (PTX3, COL6A2, CCN2, and MATN3) were verified to be upregulated in 97H cells with PBX3 overexpression compared with vector cells. And these genes were also upregulated in HLF and 97H cells with circTOLLIP overexpression (**e, f**) though qRT–PCR analysis. **g** Western blot analysis of PBX3 protein expression in circTOLLIP-overexpressing cells with or without transfection of miR-516a-5p mimic or PBX3 siRNA. **h-i** CCK-8 and colony formation assays to evaluate the cell proliferation ability respectively in four group cells. **j-l** Scratch wound healing assay and cell migration and invasion assays to evaluate the cell metastatic ability

RNA sequencing (RNA-seq) was then performed in 97H vector and PBX3- overexpressing cells (Fig. 7a). Gene set enrichment analysis (GSEA) of the target genes identified by RNA-seq to be altered upon PBX3 overexpression showed that the upregulated genes were highly enriched in EMT pathway (Fig. 7b). Consistent with this result, genes whose expression was positively correlated with high PBX3 expression were also enriched in the EMT pathway in The Cancer Genome Atlas (TCGA) database (Fig. 7c). We selected the top 4 EMT-related genes (PTX3, COL6A2, CCN2, and MATN3), which were both differentially upregulated in the RNA-seq profiles and highly correlated with high PBX3 expression in TCGA, for further investigation. qRT–PCR analysis revealed that the mRNA levels of PTX3, COL6A2, CCN2 and MATN3 were significantly increased both in PBX3-overexpressing cells and in circTOLLIP- overexpressing cells (Fig. 7d-f). This result suggested that circTOLLIP can activate EMT pathway via the miR-516a-5p/PBX3 axis.

We further investigated through rescue assays whether circTOLLIP exerts its biological effect via circTOLLIP/miR-516a-5p/PBX3 axis. The results showed that PBX3 protein expression was upregulated in circTOLLIP-overexpressing cells and that transfection of the miR-516a-5p mimic or PBX3 siRNA could attenuated the upregulation of PBX3 protein expression (Fig. 7g). Functionally, the CCK-8 assay, colony formation assay and migration and invasion assays demonstrated that ectopic overexpression of miR-516a-5p or knockdown of PBX3 could partially rescued the circTOLLIP-induced promotion of proliferation, migration, and invasion in HCC cells (Fig. 7h-l; Additional file 13: Fig. S13a-d). Collectively, these results demonstrate that circTOLLIP promotes the growth and metastasis of HCC cells through the circTOLLIP/miR-516a-5p/PBX3/EMT axis.

## Disscussion

**Fig. 8:**
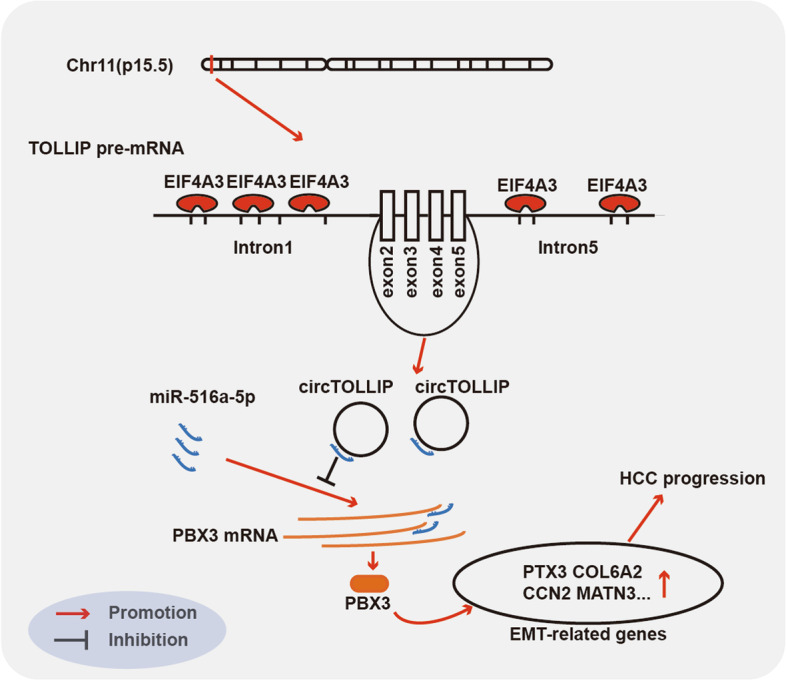
Schematic diagram of the mechanism by which circTOLLIP promotes HCC progression. EIF4A3-induced circTOLLIP promotes the expression of PBX3 by sponging miR-516a-5p, in turn activating the PBX3/EMT signaling pathway and promoting the progression of HCC

Currently, circRNAs are widely investigated as crucial molecules participating in the pathogenesis of tumorigenesis and tumor progression. Microarrays are reported to be efficient tools for profiling aberrantly expressed circRNAs in cancer [31]. Therefore, we identified the circRNAs that are abnormally expressed in HCC and matched nontumor tissues by analysis of two GEO circRNA microarray datasets. We finally focused on a novel circRNA, hsa_circ_0008301 (termed circTOLLIP), which is derived from exons of TOLLIP. It was significantly upregulated in HCC tissues and high expression of circTOLLIP positively correlated with poor prognosis in HCC patients. Functionally, we found that overexpression of circTOLLIP significantly accelerated the growth and metastasis of HCC cells in vitro and in vivo, while knockdown of circTOLLIP produced the opposite effects. Mechanistically, we demonstrated that EIF4A3 was involved in the biogenesis of circTOLLIP, which was at least one crucial cause of the formation of circTOLLIP. Furthermore, circTOLLIP functioned as a sponge of miR-516a-5p to attenuate the inhibitory effect of miR-516a-5p on PBX3, which accelerated the activation of the PBX3/EMT signaling pathway and promoted the progression of HCC (Fig. 8). However, comparing with the upregulated circ0003998 in HCC [32], whether circTOLLIP is also upregulated in metastatic tumor tissues compared to tumor tissues needs to be explored to further clarify the EMT-promoting role of circTOLLIP in HCC patients. Moreover, circ-CDYL was reported to increase the portion of epithelial cell adhesion molecule (EPCAM)-positive liver tumor-initiating cells, and a treatment combining traditional approaches and circ-CDYL interference was highly effective in inhibiting the proliferation of HCC cells [33]. Considering the metastasis-promoting mechanism of circTOLLIP in HCC, a therapeutic effect of circTOLLIP interference is also likely and could be further investigated in a model of metastatic HCC.

Considering that the exons that form circTOLLIP are part of the TOLLIP mRNA, we explored the expression of TOLLIP in HCC cells, and the results showed no significant change upon either upregulation or downregulation of circTOLLIP. This suggests that the biological functional change in HCC cells caused by circTOLLIP is unlikely to be associated with TOLLIP. The biogenesis of circRNAs is regulated by certain trans-acting factors [34], and several RNA binding proteins, such as QKI, DHX9, ADAR1, FUS and EIF4A3, have been reported to regulate the expression of circRNAs [14, 20, 21, 23, 35]. We predicted the potential binding sequence for several RNA bind-proteins in the circTOLLIP pre-mRNA through CircInteractome analysis and found five EIF4A3 binding sites in TOLLIP pre-mRNA flanking the circTOLLIP sequence. EIF4A3 was reported to be a novel component of the exon junction complex [22], which is widely involved in RNA splicing, mRNA export, and nonsense-mediated mRNA decay [36, 37]. Additionally, EIF4A3 plays an oncogenic role in several malignant tumors, including HCC [38, 39]. Separate from its regulatory role in the cell cycle and apoptosis [40], EIF4A3 can promote the expression of circRNAs by binding to pre-mRNAs [23, 41]. We confirmed that EIF4A3 promoted the expression of circTOLLIP by binding to TOLLIP pre-mRNA and that EIF4A3 did not influence the degradation of circTOLLIP. However, whether EIF4A3 participates in the transport of TOLLIP pre-mRNA and the specific mechanism by which EIF4A3 mediates pre-mRNA back-splicing need to be further clarified.

CircRNAs in the cytoplasm have widely been reported to act as microRNA sponges. We confirmed that circTOLLIP was predominantly localized in the cytoplasm and served as a sponge of miR-516a-5p, which showed lower expression in HCC tumor tissues. Functionally, we first demonstrated the tumor-suppressive role of miR-516a-5p in HCC both in vitro and in vivo and identified PBX3 as a novel target of miR-516a-5p via its binding to the 3′-UTR of the PBX3 mRNA in HCC cells. However, as reported, miR-516a-5p facilitates human bladder cancer progression by targeting PHLPP2 and inhibiting SMURF1-mediated MMP9 protein degradation [42]. This shows that the dual function of miR-516a-5p depends on the downstream target genes and the gene-associated pathways in different tumors.

PBX3 is reported to be primarily regulated by microRNAs via post-transcriptional inhibition. For example, in prostate cancer, PBX3 was found to be upregulated in prostate cancer and directly regulated by miR-let-7d [43]. Additionally, previous study has demonstrated that PBX3 can promote EMT-related gene transcription in colorectal cancer and gastric cancer [29, 44]. In colorectal cancer, PBX3 is induced by WNT activation and by the EMT transcription factors SNAIL and ZEB1. And PBX3 expression is required for a full EMT phenotype in colon cancer cells [29]. In gastric cancer, PBX3 promotes the activation of the AKT pathway, subsequently facilitating the EMT transition [44]. In HCC, PBX3 was reported to be crucial for liver tumor-initiating cells [30]. In our study, we first demonstrated through RNA-seq analysis that PBX3 can activate the EMT pathway in HCC cells and then found that PBX3 was significantly upregulated in HCC specimens and exerted an oncogenic biological effect in HCC cells. Notably, the main EMT-associated genes dysregulated upon upregulation of PBX3 in HCC were different from those dysregulated in colorectal cancer and in gastric cancer upon upregulation of PBX3, indicating that PBX3 could activate a novel EMT-related pathway in HCC. Moreover, as PBX3 is a transcription factor, whether it directly regulates the transcription of EMT-related genes in HCC needs to be further studied.

## Conclusion

In conclusion, we identified a novel upregulated circRNA termed circTOLLIP in HCC and EIF4A3 was confirmed to be involved in the biogenesis of circTOLLIP. Furthermore, our study revealed that circTOLLIP directly binds to miR-516-5p to attenuate its inhibitory effect on PBX3 expression, hence activating the PBX3/EMT signaling pathway and promoting the proliferation and metastasis of HCC cells.

## Supplementary Information

**Additional file 1.**

**Additional file 2.**

**Additional file 3.**

**Additional file 4.**

**Additional file 5.**

**Additional file 6.**

**Additional file 7.**

**Additional file 8.**

**Additional file 9.**

**Additional file 10.**

**Additional file 11.**

**Additional file 12.**

**Additional file 13.**

**Additional file 14.**

**Additional file 15.**

**Additional file 16.**

## Data Availability

All the data used in the current study is available from the corresponding authors on reasonable request.

## Abbreviations

circRNA: circular RNA
HCC: Hepatocellular carcinoma
qRT–PCR: quantitative real-time PCR
ISH: In situ hybridization
RIP: RNA immunoprecipitation
FISH: Fluorescence in situ hybridization
EIF4A3: Eukaryotic initiation factor 4A3
PBX3: PBX homeobox 3
OS: Overall survival
DFS: Disease-free survival
EMT: Epithelial to mesenchymal transition
TACE: Transarterial chemoembolization
miRNA: microRNA
MREs: MiRNA response elements
ceRNA: competing endogenous RNA
GEO: Gene expression omnibus
TOLLIP: Toll interacting protein
siRNAs: Small interfering RNAs
AGO2: Argonaute2
RAP: RNA Antisense Purification
CCK-8: Cell Counting Kit-8
cDNA: Reverse-transcribed RNA
gDNA: genomic DNA
RNA-seq: RNA sequencing
GSEA: Gene set enrichment analysis
TCGA: The Cancer Genome Atlas
