# Supplementary figures and images for: EIF4A3-induced circTOLLIP promotes the progression of hepatocellular carcinoma via the miR-516a-5p/PBX3/EMT pathway
Source: J Exp Clin Cancer Res. 2022 May 5;41:164. doi: 10.1186/s13046-022-02378-2 (PMC9069765; doi:10.1186/s13046-022-02378-2)

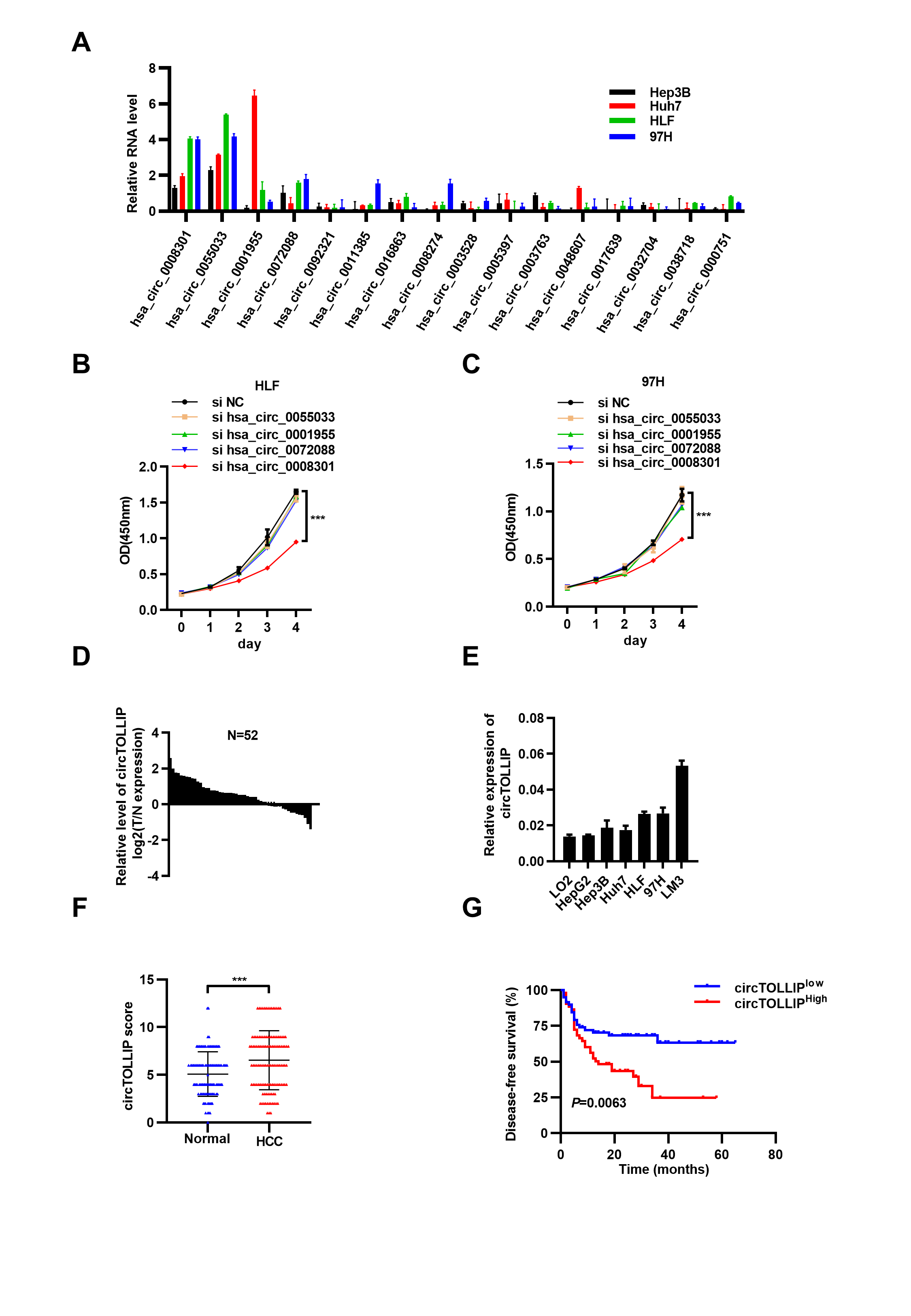

Supplement: Supplementary file 1 — Additional file 1. [file 13046_2022_2378_MOESM1_ESM.tif]

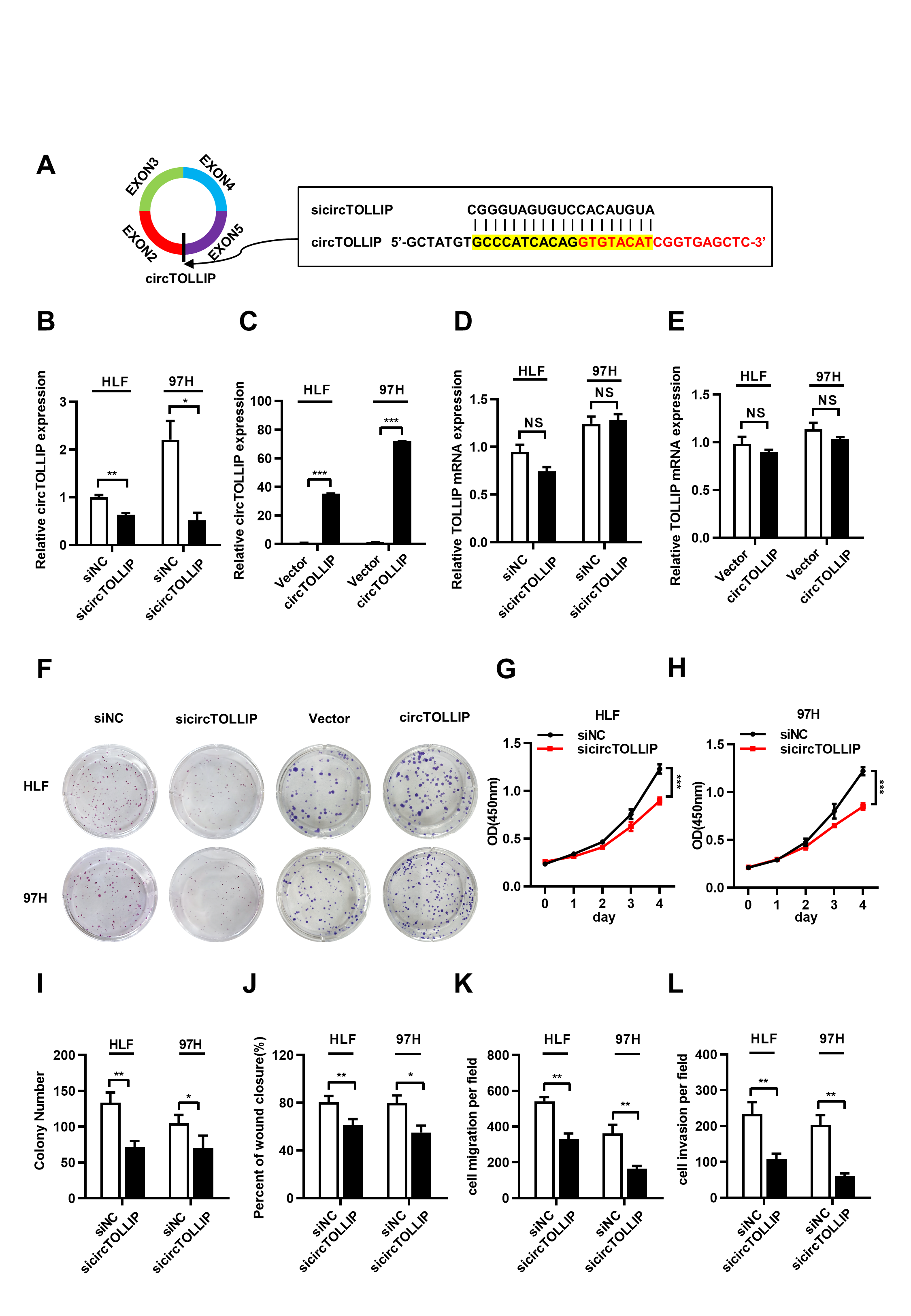

Supplement: Supplementary file 2 — Additional file 2. [file 13046_2022_2378_MOESM2_ESM.tif]

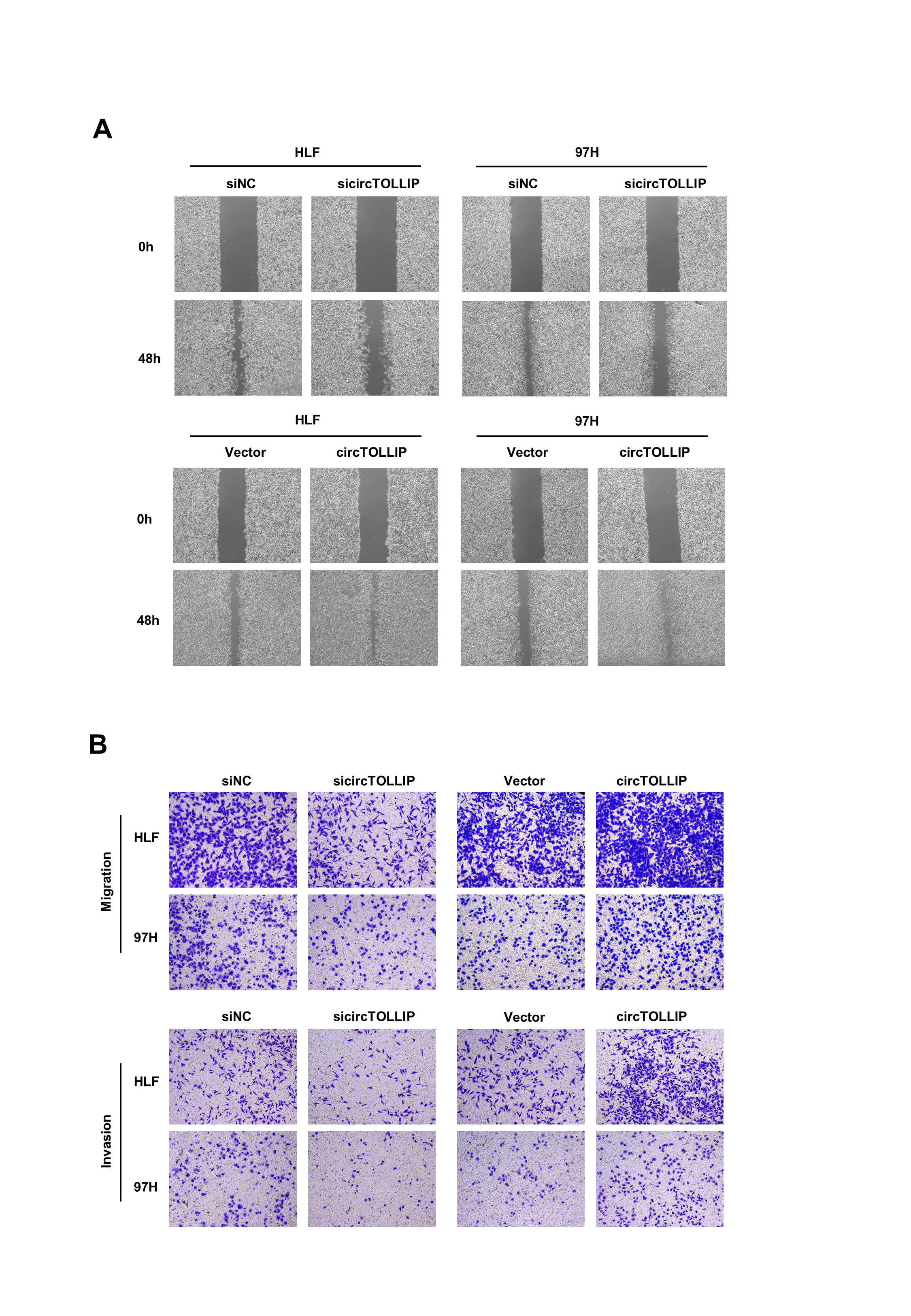

Supplement: Supplementary file 3 — Additional file 3. [file 13046_2022_2378_MOESM3_ESM.tif]

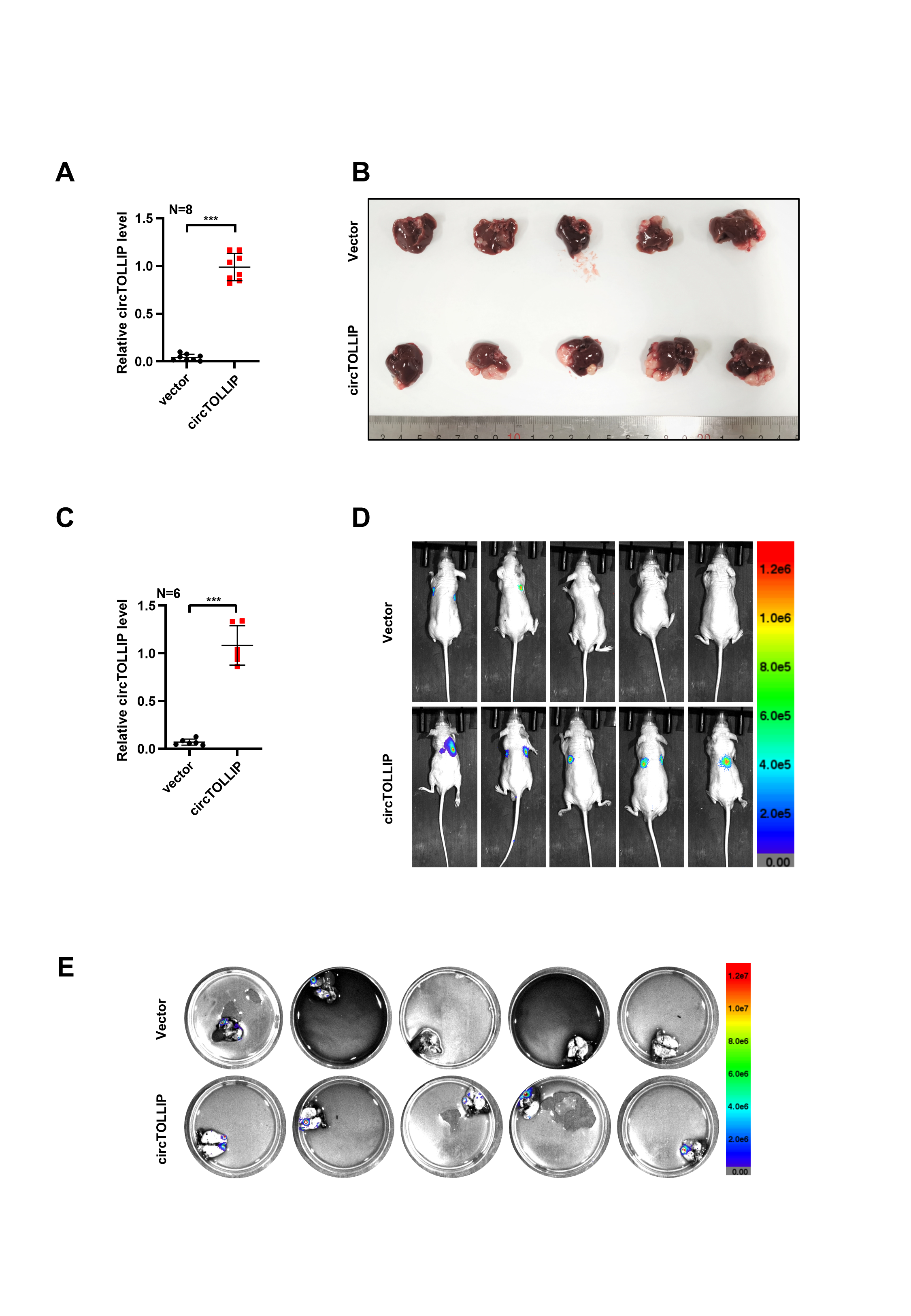

Supplement: Supplementary file 4 — Additional file 4. [file 13046_2022_2378_MOESM4_ESM.tif]

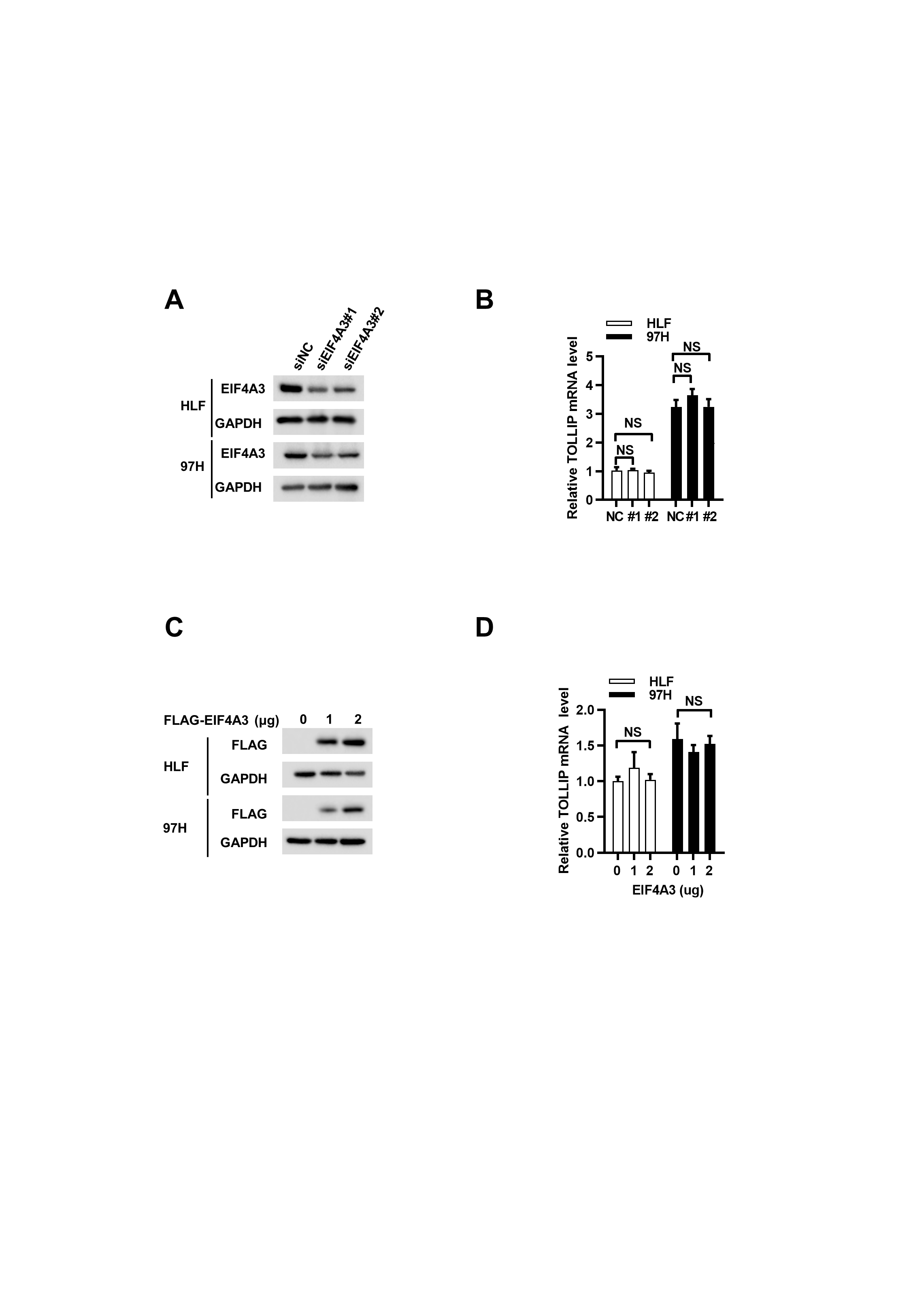

Supplement: Supplementary file 5 — Additional file 5. [file 13046_2022_2378_MOESM5_ESM.tif]

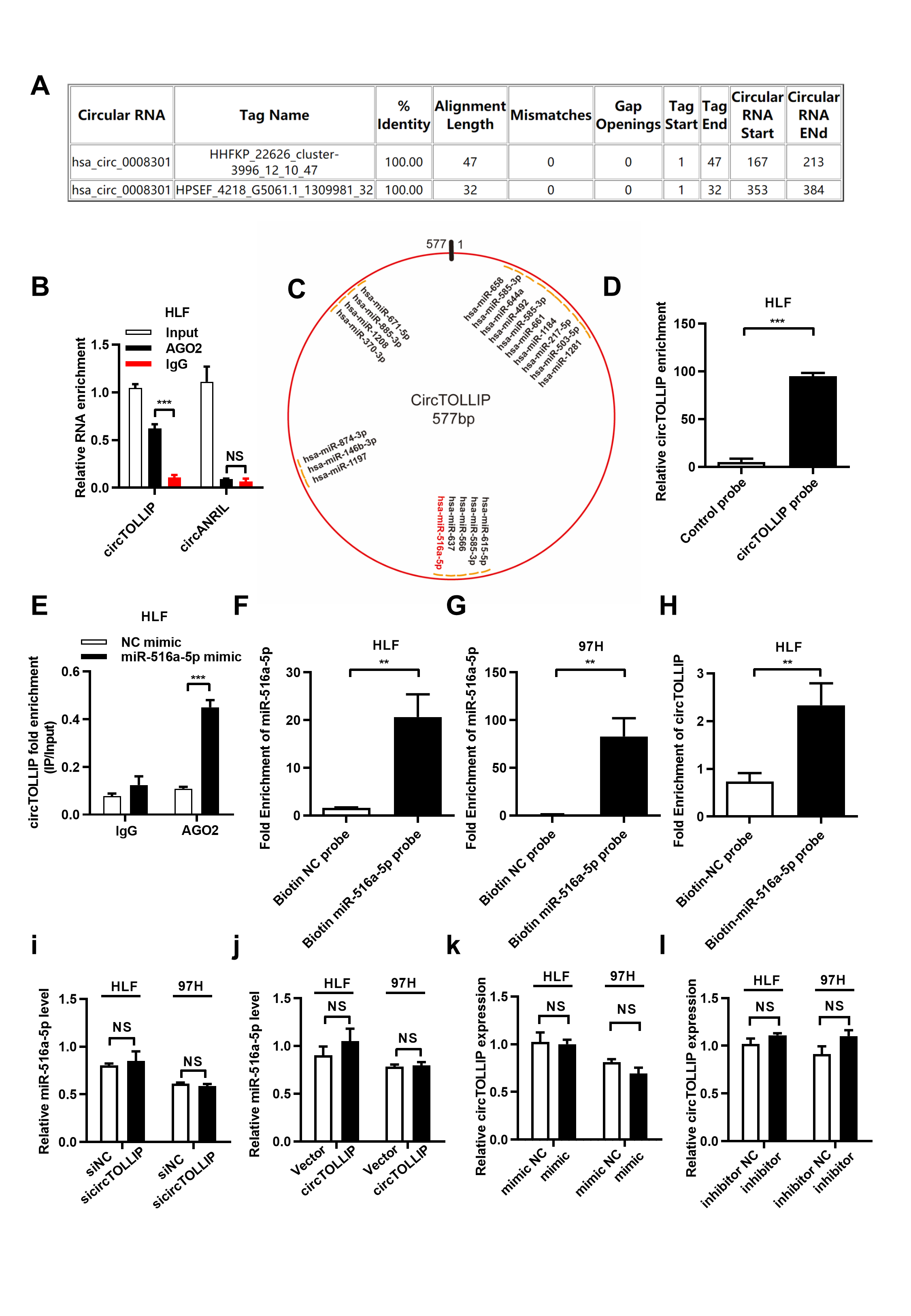

Supplement: Supplementary file 6 — Additional file 6. [file 13046_2022_2378_MOESM6_ESM.tif]

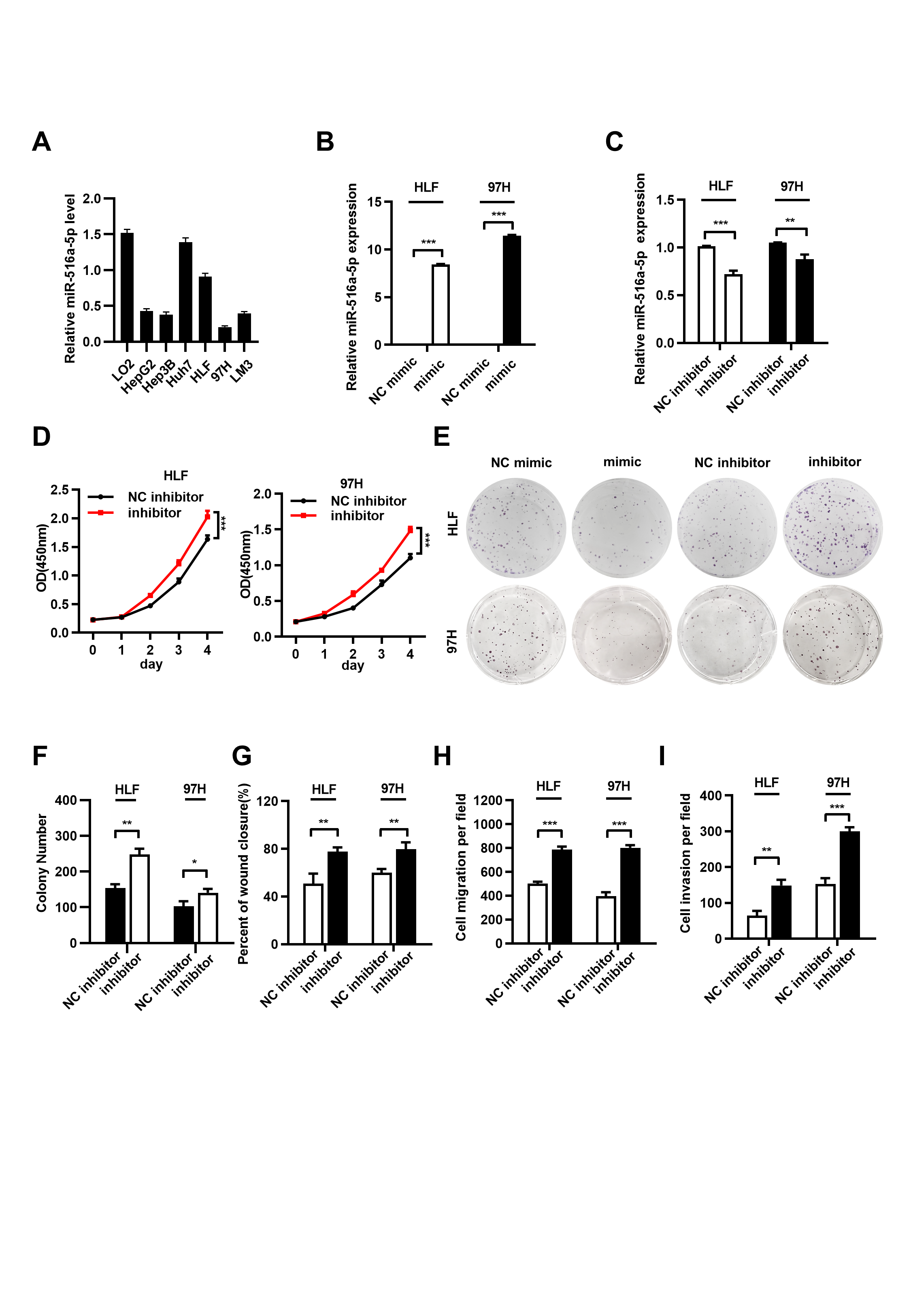

Supplement: Supplementary file 7 — Additional file 7. [file 13046_2022_2378_MOESM7_ESM.tif]

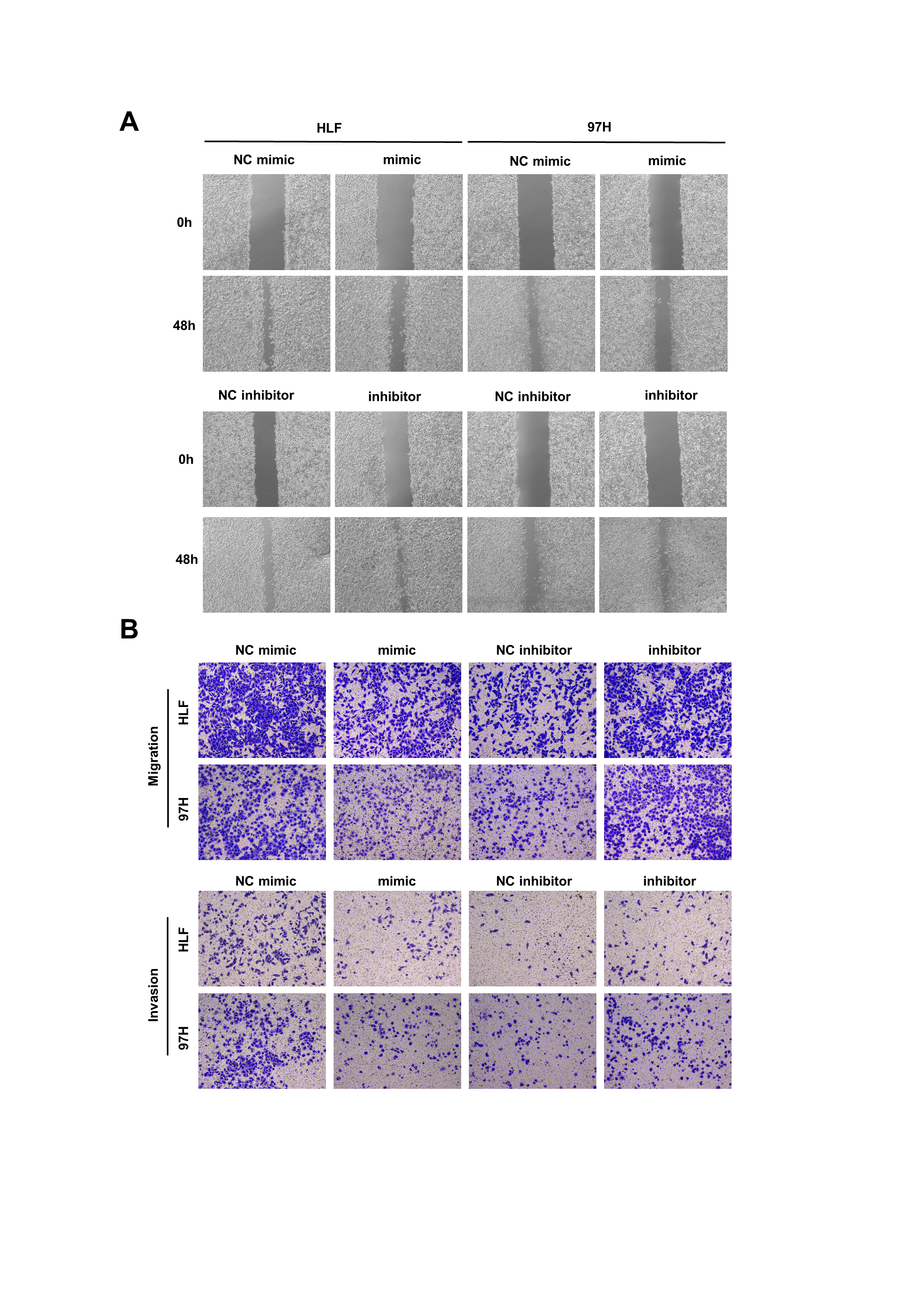

Supplement: Supplementary file 8 — Additional file 8. [file 13046_2022_2378_MOESM8_ESM.tif]

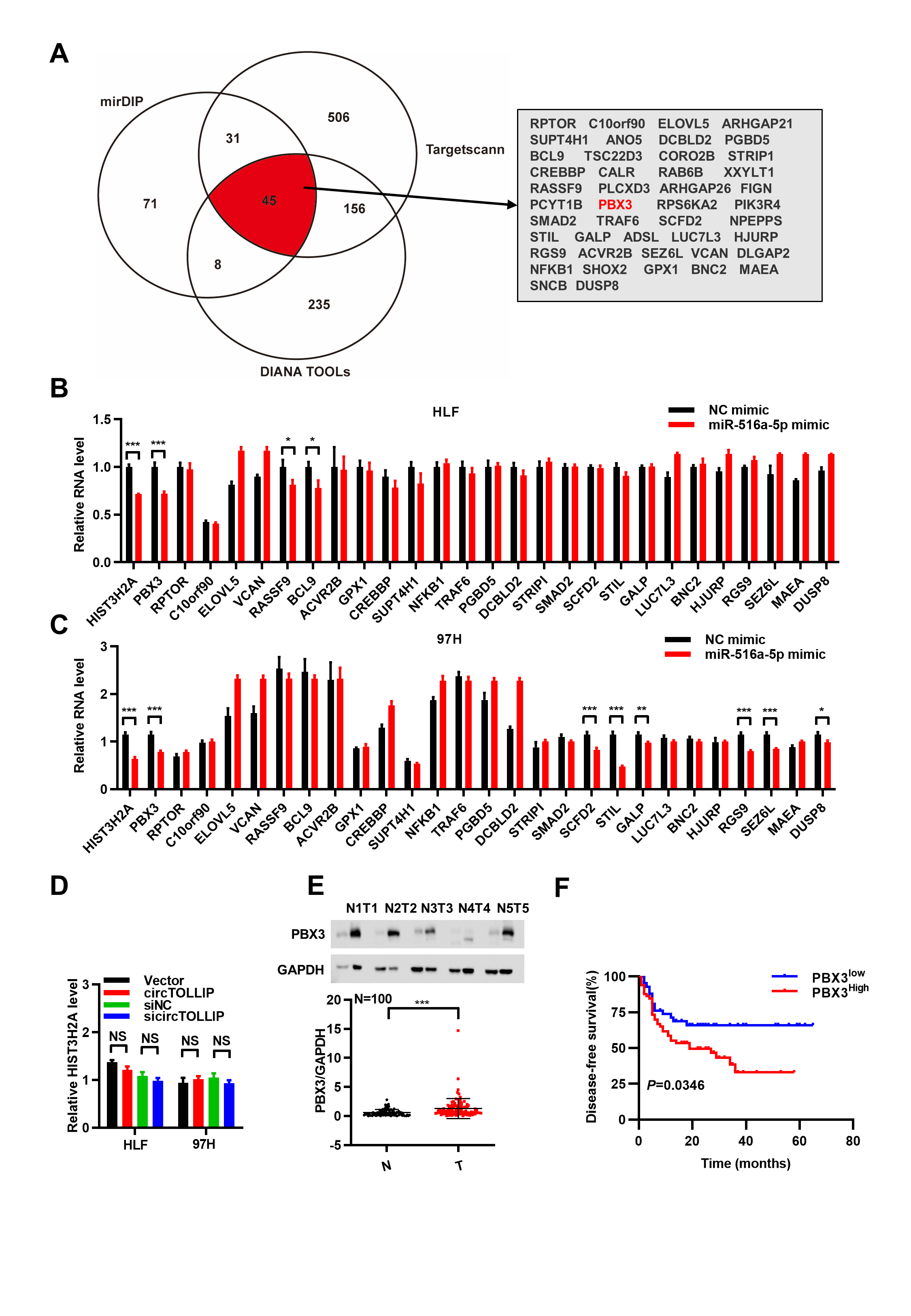

Supplement: Supplementary file 9 — Additional file 9. [file 13046_2022_2378_MOESM9_ESM.tif]

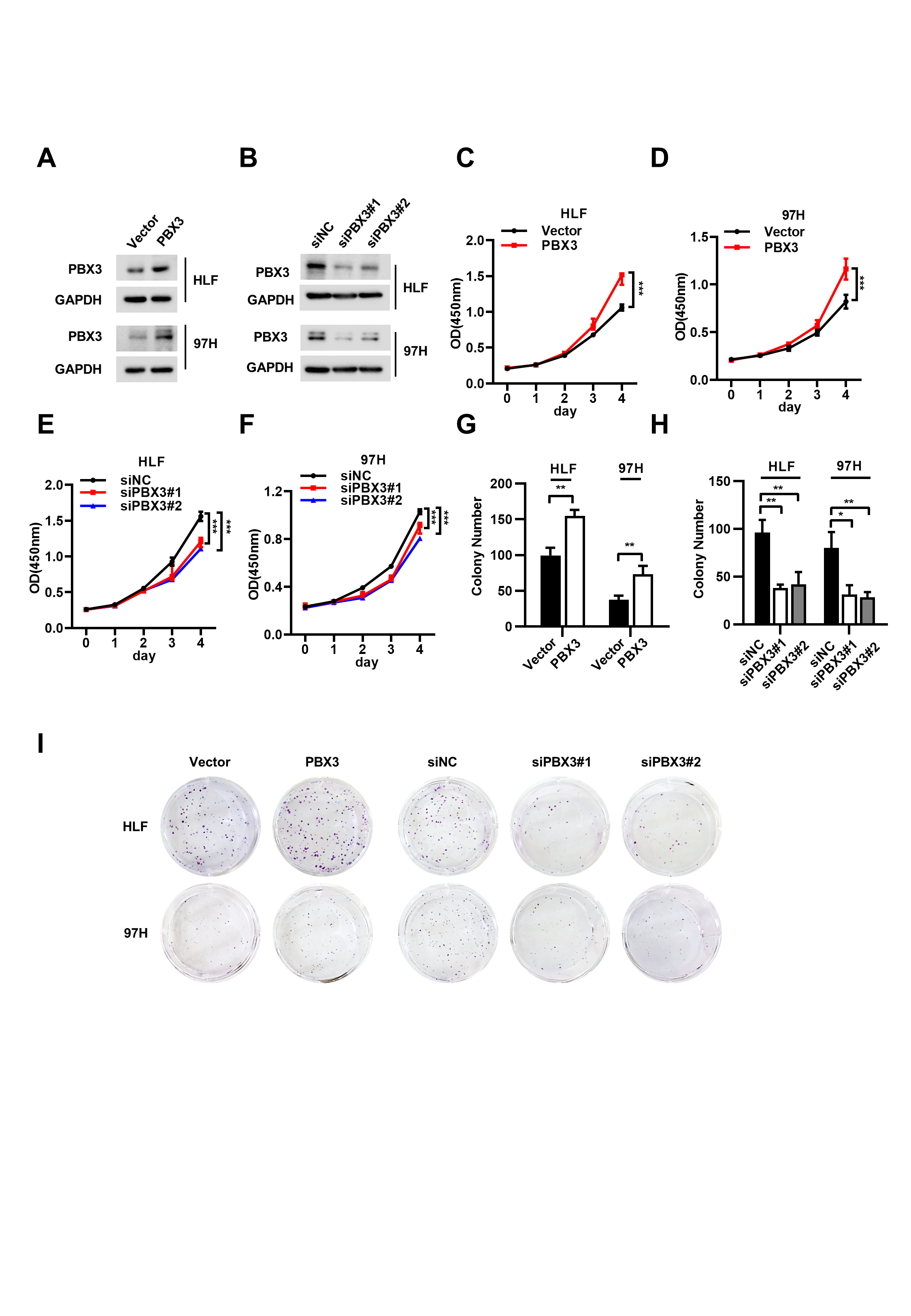

Supplement: Supplementary file 10 — Additional file 10. [file 13046_2022_2378_MOESM10_ESM.tif]

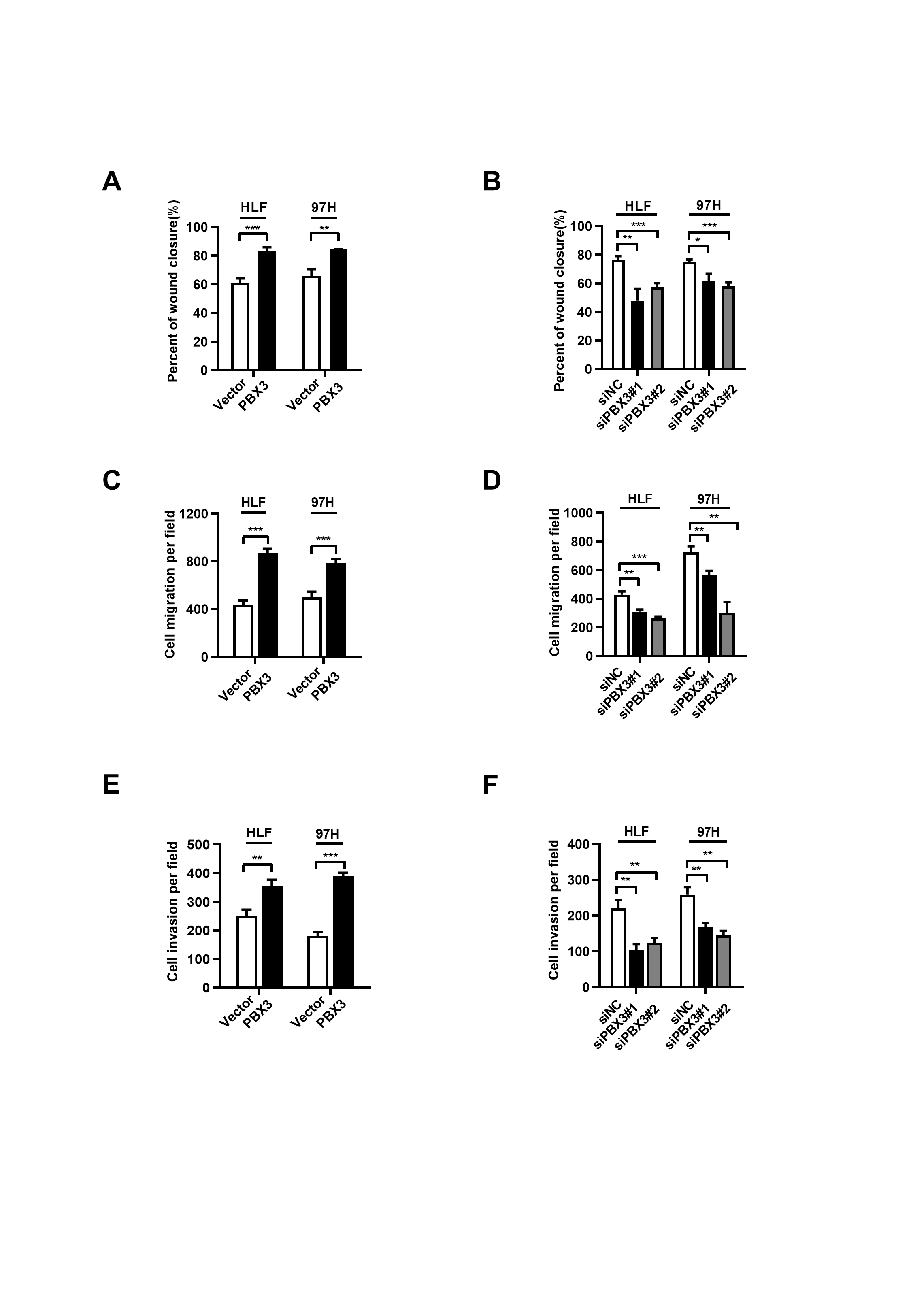

Supplement: Supplementary file 11 — Additional file 11. [file 13046_2022_2378_MOESM11_ESM.tif]

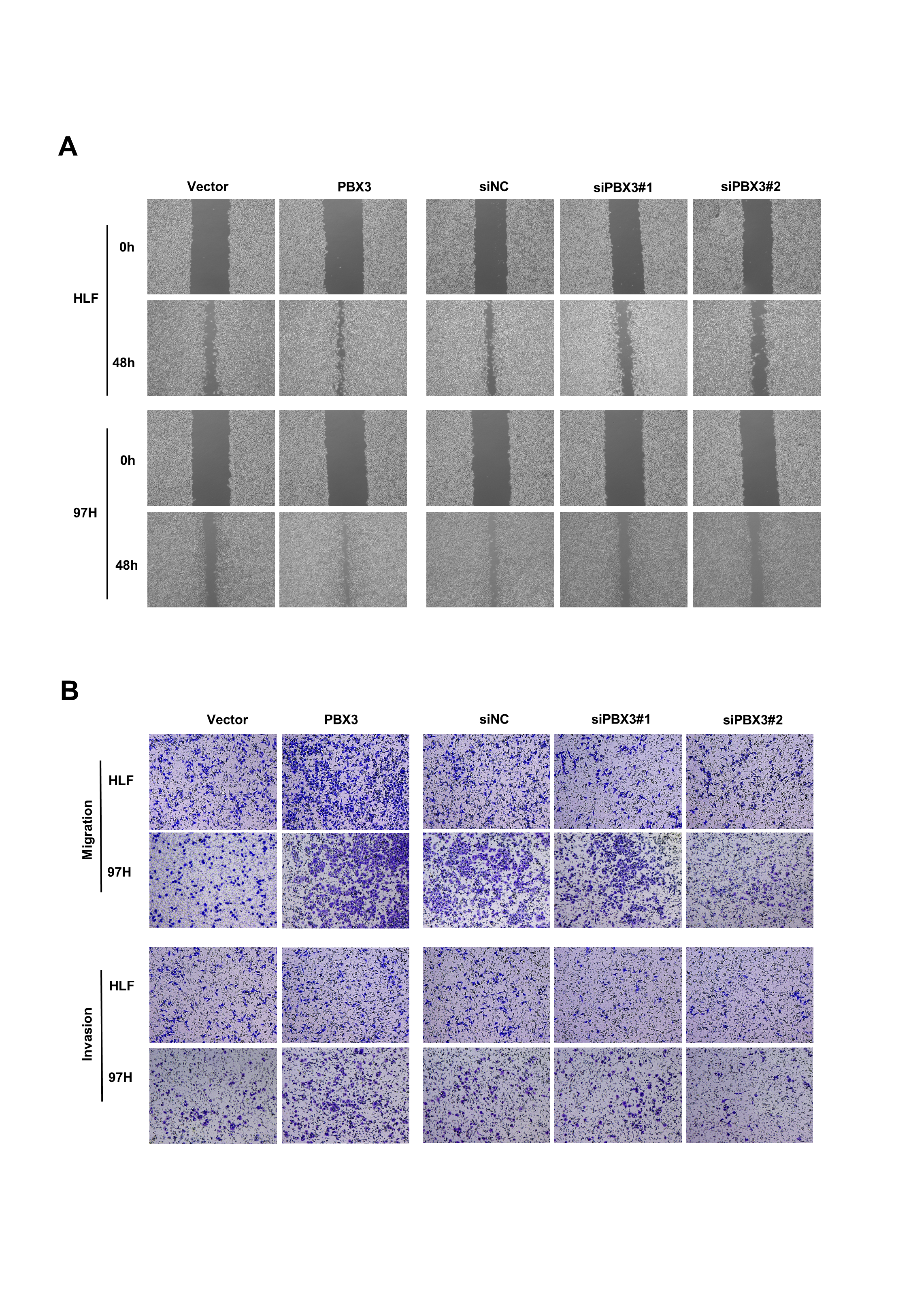

Supplement: Supplementary file 12 — Additional file 12. [file 13046_2022_2378_MOESM12_ESM.tif]

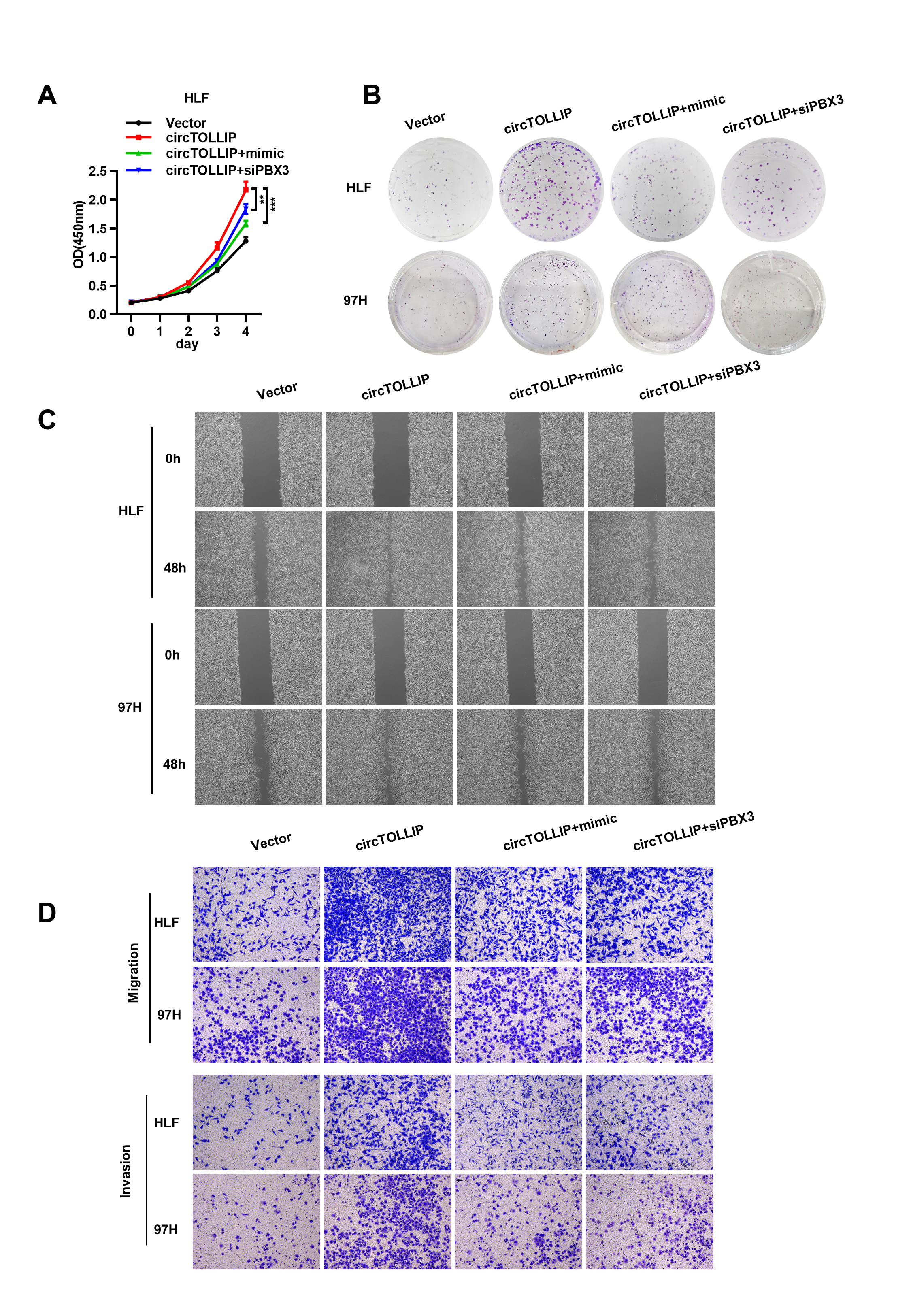

Supplement: Supplementary file 13 — Additional file 13. [file 13046_2022_2378_MOESM13_ESM.tif]
