# Supplementary material for: EIF4A3-induced circTOLLIP promotes the progression of hepatocellular carcinoma via the miR-516a-5p/PBX3/EMT pathway
Source: J Exp Clin Cancer Res. 2022 May 5;41:164. doi: 10.1186/s13046-022-02378-2 (PMC9069765; doi:10.1186/s13046-022-02378-2)
Supplement: Supplementary file 14 — Additional file 14. [file 13046_2022_2378_MOESM14_ESM.docx]

**Additional file 14: Supplementary table S1: Primers used in this study**

The primers of A, B, C, D, E and F in table S1 could amplify sequences showing in Figure 3b.

| Primer | Sequence (5’-3’) |
| --- | --- |
| circTOLLIP-divergent-F | CAGCCCGTGGTCCTGATG |
| circTOLLIP-divergent-R  circTOLLIP-convergent-F | CTGCTGTGTGGGCGTGAT  GCATGGACCCCTACTGCC |
| circTOLLIP-convergent-R | GGATGACCTTATTCCAGCGG |
| TOLLIP-F | TCAACCTCGTCATGTCCTACG |
| TOLLIP-R | TGTGATGGGCACATAGCCAAC |
| cricANRIL-F | GCTGGGATTACAGGTGTGAGACACC |
| cricANRIL-R | GAATCAGAATGAGGCTTATTCTTCTCATC |
| U6-F | CTCGCTTCGGCAGCACA |
| U6-R | AACGCTTCACGAATT TGCGT |
| GAPDH-F | TCAAGAAGGTGGTGAAGCAG |
| GAPDH-R | CGTCAAAGGTGGAGGAGTG |
| A-F | TCCTCTCTCTTCCCCTCAGA |
| A-R | CCCAGAAGAACGCAGGAAGTA |
| B-F | GTTTGACCAAGTGACCTGCA |
| B-R | TCAGGTGGGTTTTCAGGTCA |
| C-F | GATGCCAACAGTGTACCAGC |
| C-R | GGGAGCTCACCGATGTACA |
| D-F | ATTGTTGGCAGAAATGAGGTG |
| D-R | CAGAGCACGTGTCACCTGA |
| E-F | GTGTTAGGGGAGGGTCTCG |
| E-R | CCTCTCCACTCCAGCTGG |
| F-F | GTGCAGTTCATGGGGCTAGT |
| F-R | TCACAGCCTTGACGAGTGAG |
| HIST3H2A-F | GGTCGTGGTAAGCAGGGTG |
| HIST3H2A-R | CGCTCCGAATAGTTGCCCTT |
| PBX3-F | GATGCAGCTCAAACAAAGCAC |
| PBX3-R | AGTTACGCCTTTTCCGTCTGG |
| NFKB1-F | GGCAGCACTACTTCTTGACC |
| NFKB1-R | CAGCAAACATGGCAGGCTAT |
| CREBBP-F | CCTGCCACGTCACAGACTG |
| CREBBP-R | GGCCAGAGTTACTATTGAGGAGG |
| GPX1-F | CAGTCGGTGTATGCCTTCTCG |
| GPX1-R | GAGGGACGCCACATTCTCG |
| TRAF6-F | TTGCCATGAAAAGATGCAGAGG |
| TRAF6-R | AGCCTGGGCCAACATTCTC |
| BCL9-F | GGCCATACCCCTAAAGCACTC |
| BCL9-R | CGGAAATACTTCGCTCCCTTTT |
| VCAN-F | GCAAGTGATGCGGGTCTTTAC |
| VCAN-R | TTGCCGCCCTGTAGTGAAAC |
| ACVR2B-F | TCAAGGGGAACATCATCACA |
| ACVR2B-R | TCTGTGAATGTTGACTCCGCT |
| C10orf90-F | TGTGCCATTGCTCAGTCTCG |
| C10orf90-R | TCTGTGAATGTTGACTCCGCT |
| RASSF9-F | ACAACAATCCCGCAGTTCAAA |
| RASSF9-R | GTGTCTGGATTTCCAGGGTGA |
| DCBLD2-F | ATGTGGACACACTGTACTAGGC |
| DCBLD2-R | CTGTTGGGATAGGTCTGTGGG |
| ELOVL5-F | TAACAGGAGTATGGGAAGGCA |
| ELOVL5-R | ACCAGAGGACACGGATAATCTT |
| RPTOR-F | ACTGGAACCTACCTTTGGCTT |
| RPTOR-R | ACTGTCTTCATCCGATCCTTCA |
| SUPT4H1-F | ACCGAGAGATGGTATATGACTGC |
| SUPT4H1-R | TGGCTTAAAGTTACTGACTCGC |
| PGBD5-F | CAGCCAAGCGATTCATTCACA |
| PGBD5-R | CACCATGTTCTTGAGGACGTT |
| STRIP1-F | GCTCTCGGAGCTTTACAGCTA |
| STRIP1-R | CCACTTCTTGTCTGTCACATGGA |
| SMAD2-F | CCGACACACCGAGATCCTAAC |
| SMAD2-R | GAGGTGGCGTTTCTGGAATATAA |
| SCFD2-F | GGACCGTGGAGATCCTACG |
| SCFD2-R | CGCCGCTGGGACATGATTA |
| STIL-F | GAAGACCATCCGACTTGCTTATC |
| STIL-R | GGATCAAAGCGATCTACTGTCAA |
| GALP-F | CTGAGCCTGGCAGAGACTC |
| GALP-R | TCTAGGATCTCAAGGGCTGTC |
| LUC7L3-F | GACCACGAGAGCGTTTGTAAA |
| LUC7L3-R | CGGACCAAGATCAGAACGTGTAT |
| BNC2-F | ACAACTCCATGCAGTTCGGAA |
| BNC2-R | GATGGCCTGTTGGGACATTCT |
| HJURP-F | CACAAAGCCATCAAGCATCATC |
| HJURP-R | TCAGAGCAGGGTATGAAGTTCT |
| RGS9-F | TTCAGCGAATTGATCCGAGAC |
| RGS9-R | TCCATACTTCAGATCCTCGCA |
| MAEA-F | CTCCCCGTACAAGGACCTTCT |
| MAEA-R | GGTGTAGTCGGTAGTTGTCGTA |
| SEZ6L-F | GAGCGAGATGCTCTTCCCG |
| SEZ6L-R | GGCGCTGTTACCACTCTCT |
| DUSP8-F | GTCCCCATCAACGACAACTAC |
| DUSP8-R | CAGTGGACGATGACTTGGCAG |
| COL6A2-F | GACTCCACCGAGATCGACCA |
| COL6A2-R | CTTGTAGCACTCTCCGTAGGC |
| PTX3-F | AGGCTTGAGTCTTTTAGTGCC |
| PTX3-R | ATGGATTCCTCTTTGTGCCATAG |
| MATN3-F | ACATGGCGTCCCTCAAGATG |
| MATN3-R | GCACAGAAGGTTTCCTGGAATC |
| CCN2-F | AAAAGTGCATCCGTACTCCCA |
| CCN2-R | CCGTCGGTACATACTCCACAG |
| hsa-miR-146b-3p-F | TGCCCTGTGGACTCAGTTCTG |
| hsa-miR-885-3p-F | AGGCAGCGGGGTGTAGT |
| hsa-miR-874-3p-F | CTGCCCTGGCCCGAGGG |
| hsa-miR-671-5p-F | AGGAAGCCCTGGAGGGG |
| hsa-miR-661-F | TGCCTGGGTCTCTGGCC |
| hsa-miR-658-F | GGCGGAGGGAAGTAGGTCC |
| hsa-miR-644a-F | CGGTCAGTGTGGCTTTCTTAGAGC |
| hsa-miR-637-F | ATACTGGGGGCTTTCGGGC |
| hsa-miR-615-5p-F | ATGGGGGTCCCCGGTGCTC |
| hsa-miR-585-3p-F | CGCTGGGCGTATCTGTATGCTA |
| hsa-miR-566-F | GGGCGCCTGTGATCCCAA |
| hsa-miR-516a-5p-F | CCGTTCTCGAGGAAAGAAGCACTTTC |
| hsa-miR-503-5p-F | TAGCAGCGGGAACAGTTCTGC |
| hsa-miR-492-F | AGGACCTGCGGGACAAGATTCTT |
| hsa-miR-370-3p-F | GCCTGCTGGGGTGGAAC |
| hsa-miR-217-5p-F | CCGTACTGCATCAGGAACTGATTGGA |
| hsa-miR-1281-F | TCGCCTCCTCCTCTCCC |
| hsa-miR-1208-F | GTCACTGTTCAGACAGGCGGA |
| hsa-miR-1197-F | CGCGTAGGACACATGGTCTACTTCT |
| hsa-miR-1184-F | CCTGCAGCGACTTGATGGC |
