# Supplementary material for: EIF4A3-induced circTOLLIP promotes the progression of hepatocellular carcinoma via the miR-516a-5p/PBX3/EMT pathway
Source: J Exp Clin Cancer Res. 2022 May 5;41:164. doi: 10.1186/s13046-022-02378-2 (PMC9069765; doi:10.1186/s13046-022-02378-2)
Supplement: Supplementary file 16 — Additional file 16. [file 13046_2022_2378_MOESM16_ESM.docx]

**Supplementary Information**

**Supplementary Methods**

**Cellines and cell culture**

The 7702, HepG2, Hep3B, Huh7, HLF, and HEK-293T cells were purchased from the China Center for Type Culture Collection (CCTCC, Wuhan, China). The HCC cell lines LM3 and 97H were obtained from the Liver Cancer Institute of Fudan University. All cell lines were cultured in Dulbecco’s modified Eagle’s medium (DMEM) supplemented with 10% fetal bovine serum (Gibco, USA) and incubated in a 5% CO2 atmosphere at 37 °C.

**qRT–PCR**

Total RNA was extracted using TRIzol reagent (Takara, Japan). A PARIS Kit (Ambion, Life Technologies) was used for the nuclear and cytoplasmic RNA extraction. Reverse transcription was conducted with HiScriptII Q RT SuperMix for qPCR (Vazyme) or Mir-X miRNA First-Strand Synthesis Kit (Takara, Japan). qRT–PCR was performed using ChamQ Universal SYBR qPCR Master Mix (Vazyme) in a CFX Connect™ Real-Time PCR Detection System (Bio–Rad, USA). U6 and GAPDH were used as the controls. The relative expression levels of mRNA, circRNA and microRNA were calculated using the comparative CT (2-^ΔΔCT^) method. All reactions were repeated three times independently. The primers are listed in Additional file 14: supplementary table S1.

***In situ* hybridization and immunohistochemical (IHC) staining**

The expression of circTOLLIP in formalin-fixed, paraffin-embedded clinical samples was assessed by ISH. A digoxin-coupled circTOLLIP probe (5’-digoxin- CTGCGGGAGCTCACCGATGTACACCTGTGATGGGCACATAGCCAACGC) was synthesized by Sangon Biotech. ISH was performed using Enhanced Sensitive ISH Detection Kit I (POD) (BOSTER, China) according to the manufacturer’s protocol. The expression of EIF4A3 and PBX3 was evaluated by IHC staining. IHC staining was conducted on formalin-fixed, paraffin-embedded tissue sections by using the polymer HRP detection system (Zhongshan Goldenbridge Biotechnology, China). Briefly, tissue sections were deparaffinized in xylene, rehydrated with ethanol and subjected to antigen retrieval in boiling citrate buffer for 15 min, followed by incubation with 5% BSA to block non-specific antigen binding. Primary antibodies were then incubated at 4 ℃ overnight. HRP conjugated secondary antibody was then incubated at 37 ℃ for 45 minutes. The peroxidase reaction was detected with diaminobenzidine (DAB) to evaluate the antibody-binding intensity and location. The ISH and IHC staining scores were assessed based on the percentage of stained cells (0–5% = 0, 5–25% = 1, 26–50% = 2, 51–75% = 3 and 76–100% = 4) and the staining intensity in tumor cells (none = 0, weak = 1, intermediate = 2, and strong = 3). A total score of≥6 was defined as high expression level. The patients’ data showed in supplementary table S2 in Additional file 15.

**Actinomycin D assay**

HLF and 97H cells were exposed to 2 µg/ml actinomycin D (Sigma). Total RNA was then extracted and qRT–PCR was performed to analyze the stability of circTOLLIP and TOLLIP mRNA.

**RNase R treatment**

Total RNA (5 μg) was incubated with or without 3U/μg RNase R (Geneseed, Guangzhou, China) at 37 ℃ for 15 min. Reverse transcription was subsequently conducted after digestion, then analyzed through qRT–PCR and DNA gel electrophoresis.

**Cell proliferation assay**

The cell viability was evaluated with a Cell Counting Kit-8 (CCK-8; Dojindo, Tokyo, Japan) assay and a cell colony formation assay. For the CCK-8 assay, HLF and 97H cells (1×10^3^ cells/well) were cultured in 96-well plates. At the indicated time points, the CCK-8 solution was added and incubated for 1 hour at 37℃ and the optical density (OD) was measured using a microplate reader (Bio-Tek Instruments, USA) at 450 nm. For the cell colony formation assay, HLF and 97H cells (1×10^3^ cells/well) were cultured in 6-well plates for 2 weeks. All experiments were repeated three times.

**Cell migration and invasion assays**

Transwell chambers (8 μm pore size, Corning, USA) were used for cell migration and invasion assays. For the invasion assay, the chambers were precoated with 40 μl of a 1:6 mixture of Matrigel (BD Biosciences, CA, USA) and DMEM at 37 ℃ for 1 hour. Cells (3×10^4^ 97H cells or 2×10^4^ HLF cells) were cultured in the upper chambers in DMEM. And the lower chambers contained 600 μl DMEM supplemented with 10% FBS. After incubation for 24 h at 37 °C, nonmigrated or noninvaded cells were removed mechanically. After fixing with 4% paraformaldehyde, cells on the lower surface of the membranes were stained with 0.1% crystal violet. All experiments were performed three times.

**Wound healing assay**

Cells were scratched a line with a 10 μl pipette tip when growing to 95% confluence and monolayer in 6-well plate. The images were captured at 0h, 48h after the scratch. Images of 3 random fields among three replicate wells were chosen for quantitative analysis.

**Western blot (WB)**

Total protein of HCC cells and tissues was extracted using RIPA Buffer with addition of 1% protease and 1% phosphatase inhibitor cocktail. Sodium dodecyl sulfate-polyacrylamide gel electrophoresis (SDS-PAGE) was performed to separate the boiled proteins and the separated proteins were then transferred onto polyvinylidene fluoride (PVDF) membranes. After blocking with 5% bovine serum albumin (BSA) at 37 ℃ for 1 h, the membranes were incubated first with primary antibodies against PBX3 (Abcam), EIF4A3 (Abcam), and GAPDH (Proteintech, China) overnight at 4 ℃ and followed by incubation of secondary antibody (Aksomics, China) at 37 ℃ for 1 h. Bands were detected by the ECL Detection System (Bio–Rad, USA).

**Dual-luciferase reporter assay**

HEK-293T and 97H cells were cultured in 24-well plates and were contransfected with a mixture of 100 ng luciferase reporter, 25 nM miR-516a-5p mimic, 50 nM miR-516a-5p inhibitor or 25 nM circTOLLIP siRNA using Lipofectamine 3000 reagent. 36-48h later, the cells were lysed and the Renilla and firefly luciferase activities were measured using the Dual-Luciferase Reporter Assay System (E1910, Promega). The Renilla luciferase activity was normalized to firefly luciferase activity.

**Animal experiments**

Male BALB/c nude mice (4 weeks old) were purchased from Huafukang Bioscience Co. Inc. (Beijing, China). All nude mice were housed under specific pathogen free (SPF) conditions. All animal experiments were carried out in line with the National Institutes of Health guidelines and were approved by the Ethics Committee of Tongji Hospital, HUST.

To establish the subcutaneous xenograft model, 1×10^6^ cells were injected into the axillae of nude mice and the nude mice were sacrificed 3 weeks later. To establish the orthotopic xenograft tumor metastasis model, 20 μl of Matrigel containing 1×10^6^ cells was injected intrahepatically with a 27-gauge needle, and the mice were sacrificed 4 weeks after injection. To establish the pulmonary metastasis model, 1×10^6^ cells were injected into the tail veins of nude mice, and the mice were sacrificed 6 weeks later. All mice were euthanized by anesthesia overdose.

**Supplementary Figure Legends**

**Figure.S1 Expression of circTOLLIP in HCC. a** qRT–PCR analysis of 16 circRNAs in four HCC cell lines (Hep3B, Huh7, HLF, and 97H). **b-c** CCK-8 assay in HLF and 97H cells transfected with siRNAs of 4 circRNAs. **d** Relative expression of circTOLLIP level in 52 paired HCC tissues. **e** circTOLLIP level in HCC cell lines. **f** Quantitative analysis of circTOLLIP in ISH staining results. **g** Kaplan–Meier analysis between circTOLLIP expression and disease-free survival. low circTOLLIP group: n=59, high circTOLLIP group: n=61.

**Figure.S2 CircTOLLIP promotes the proliferation and metastasis of HCC cells *in vitro.* a** Schematic diagram of circTOLLIP siRNA target sites specific to the back-splicing junction. **b-e** qRT–PCR analysis of circTOLLIP and TOLLIP mRNA level after overexpressing or silencing circTOLLIP. **f, i** Cell colony formation assay and CCK-8 assay (**g**, **h**) after transfection of circTOLLIP siRNA. **j-l**. The statistic graphs of scratch wound healing assay and cell migration and invasion assays in HLF and 97H cells transfected with circTOLLIP siRNA.

**Figure.S3 CircTOLLIP promotes the metastasis of HCC cells *in vitro.* a-b** Representative images of wound healing assays and cell migration and invasion assays after overexpressing or knocking down circTOLLIP expression.

**Figure.S4 CircTOLLIP promotes the proliferation and metastasis of HCC *in vivo.***

**a** qRT–PCR analysis of circTOLLIP level in isolated subcutaneous tumor tissues. **b** The whole liver of nude mice orthotopic transplanted with vector or circTOLLIP overexpression HLF cells. **c** qRT–PCR analysis of circTOLLIP level in liver tissues from mice of orthotopic transplantation model. **d-e** Pictures of lung fluorescence *in vivo* and *in vitro* imaging in mice of lung metastasis model.

**Figure.S5 EIF4A3 promotes the biogenesis of circTOLLIP. a** EIF4A3 protein level

in HLF and 97H cells with transfection of EIF4A3 siRNA**. b** qRT–PCR analysis of TOLLIP mRNA level in HLF and 97H cells transfected with EIF4A3 siRNA. **c** EIF4A3 protein expression in HLF and 97H cells transfected with EIF4A3 plasmids**. d** qRT–PCR analysis of TOLLIP mRNA level in HLF and 97H cells transfected with EIF4A3 plasmids.

**Figure.S6 circTOLLIP serves as a sponge for miR-516a-5p. a** Potential binding sites for AGO2 with circTOLLIP predicted by CircInteractome. **b** AGO2 RIP in HLF cells. **c** Potential microRNAs that directly binding to circTOLLIP. **d** Biotinylated circTOLLIP-probe RNA pulldown showed that circTOLLIP was successfully enriched. **e** AGO2 RIP in HLF cells with transfection of NC mimic or miR-516a-5p mimic. **f-h** Biotinylated miR-516a-5p-probe RNA pulldown showed that both miR-516a-5p and circTOLLIP were successfully enriched in HLF and 97H cells. **i-j** Relative miR-516a-5p expression after overexpressing or silencing circTOLLIP in HLF and 97H cells through qRT–PCR analysis. **k-l** Relative circTOLLIP level in HLF and 97H cells transfected with miR-516a-5p mimic or inhibitor through qRT–PCR analysis.

**Figure.S7 miR-516a-5p inhibits the proliferation and metastasis of HCC cells a** Expression of miR-516a-5p in HCC cell lines. **b-c** miR-516a-5p expression was successfully upregulated and inhibited in HLF and 97H cells. And CCK-8 (**d**) assay and cell colony formation assay (**e, f**) were performed to evaluate the cell proliferation ability. **g-i** The statistical graphs of the scratch wound healing assay and cell migration and invasion assay results after inhibiting miR-516a-5p.

**Figure.S8 miR-516a-5p inhibits the metastasis of HCC cells** **a-b** Representative pictures of the wound healing assay and cell migration and invasion assays after upregulating or inhibiting miR-516a-5p.

**Figure.S9 PBX3 is a direct target of miR-516a-5p a** Venn diagram of miR-516a-5p downstream target genes predicted from three databases. **b-c** Relative mRNA level of predicted genes in HLF and 97H cells transfected with NC mimic and miR-516a-5p mimic. **d** HIST3H2A mRNA level in HLF and 97H cells with circTOLLIP overexpression and knockdown. **e** PBX3 protein level in HCC tissues. N: nontumor tissue; T: tumor tissue. **f** The correlation between PBX3 expression and DFS through Kaplan–Meier analysis. low PBX3 group: n=42, high PBX3 group: n=66.

**Figure.S10 PBX3 promotes the proliferation of HCC cells a-b** The PBX3 protein was successfully overexpressed and knocked down in HLF and 97H cells. CCK-8 assay (**c-f**) and colony formation assay (**g-i**) in HLF and 97H cells with PBX3 overexpression or knockdown.

**Figure.S11 PBX3 promotes the metastasis of HCC cells a-f** Statistical graphs of scratch wound healing assay and cell migration and invasion assay results in HLF and 97H cells with PBX3 overexpression or knockdown.

**Figure.S12 PBX3 promotes the metastasis of HCC cells** **a-b** Representative pictures of the scratch wound healing assay and cell migration and invasion assays after upregulation or silencing of PBX3.

**Figure.S13 Upregulating miR-516a-5p or silencing PBX3 rescued the promotive effects of circTOLLIP on tumor progression.** CCK-8 assay in HLF cells (**a**) and representative images of the colony formation assay (**b**), scratch wound healing assay (**c**) and cell migration and invasion assays (**d**) in circTOLLIP-overexpressing cells with or without transfection of the miR-516a-5p mimic or PBX3 siRNA.
